# The association between early life antibiotic exposure and the gut resistome of young children: a systematic review

**DOI:** 10.1080/19490976.2022.2120743

**Published:** 2022-10-26

**Authors:** Rebecca M. Lebeaux, Despina B. Karalis, Jihyun Lee, Hanna C. Whitehouse, Juliette C. Madan, Margaret R. Karagas, Anne G. Hoen

**Affiliations:** a Department of Epidemiology, Geisel School of Medicine at Dartmouth, Hanover, NH, USA; b Department of Microbiology & Immunology, Geisel School of Medicine at Dartmouth, Hanover, NH, USA; c Program in Quantitative Biomedical Sciences, Geisel School of Medicine at Dartmouth, Hanover, NH, USA; d Department of Pediatrics, Children’s Hospital at Dartmouth, Lebanon, NH, USA; e Children’s Environmental Health & Disease Prevention Research Center at Dartmouth, Hanover, NH, USA; f Department of Biomedical Data Science, Geisel School of Medicine at Dartmouth, Hanover, NH, USA

**Keywords:** Antibiotics, children, resistome, antibiotic resistance genes, systematic review

## Abstract

Antimicrobial resistance is a growing public health burden, but little is known about the effects of antibiotic exposure on the gut resistome. As childhood (0–5 years) represents a sensitive window of microbiome development and a time of relatively high antibiotic use, the aims of this systematic review were to evaluate the effects of antibiotic exposure on the gut resistome of young children and identify knowledge gaps. We searched PubMed, Scopus, Web of Science, and the Cochrane Central Register of Controlled Trials. A PICO framework was developed to determine eligibility criteria. Our main outcomes were the mean or median difference in overall resistance gene load and resistome alpha diversity by antibiotic exposure groups. Bias assessment was completed using RoB 2 and ROBINS-I with quality of evidence assessed via the GRADE criteria. From 4885 records identified, 14 studies (3 randomized controlled trials and 11 observational studies) were included in the qualitative review. Eight studies that included information on antibiotic exposure and overall resistance gene load reported no or positive associations. Inconsistent associations were identified for the nine studies that assessed resistome alpha diversity. We identified three main groups of studies based on study design, location, participants, antibiotic exposures, and indication for antibiotics. Overall, the quality of evidence for our main outcomes was rated low or very low, mainly due to potential bias from the selective of reporting results and confounding. We found evidence that antibiotic exposure is associated with changes to the overall gut resistance gene load of children and may influence the diversity of antimicrobial resistance genes. Given the overall quality of the studies, more research is needed to assess how antibiotics impact the resistome of other populations. Nonetheless, this evidence indicates that the gut resistome is worthwhile to consider for antibiotic prescribing practices.

## Introduction

In 2019, antimicrobial resistance (AMR) was the twelfth leading cause of death globally with 1.27 million attributed deaths,^1^ making it a rapidly-growing problem.^2,3^ Many factors contribute to the increasing burden of AMR including the use and misuse of antibiotics in agriculture and humans.^4,5^ Many studies focus on phenotypic AMR using microbiological assays,^1,6^ but this ignores underlying antimicrobial resistance genes (ARGs) in human, animal, or environmental resistomes that can lead to antibiotic-resistant infections.^7^ Likewise, information on pathogenic antibiotic-resistant organisms is important, but ignores commensal and possibly pathogenic organisms that have the potential to transfer ARGs and provide colonization resistance to the gut microbiota.^8^ As there are many opportunities to mitigate these impacts in humans including preventing transmission and selection of ARGs through hospital and community spread,^1^ encouraging vaccination to decrease direct and indirect infection to resistant organisms,^9^ delaying antibiotic prescribing in favor of watchful waiting,^10^ and developing new microbiome-based therapeutics that can prevent the recurrence of antibiotic-resistant infections,^11^ measuring the collection of all ARGs in microbial communities (resistomes) is crucial.

The largest reservoir for ARGs in humans is in the gut.^12^ Perturbations to the gut microbiome have been associated with numerous health concerns including loss of colonization resistance,^8,13^ an increase in potentially pathogenic antibiotic-resistant organisms,^14^ and reduced microbiome resiliency due to decreases in strain-level diversity.^15,16^ Systematic literature reviews have attempted to assess the human gut resistome,^17,18^ but persistent gaps remain, including our understanding of how perturbations lead to alterations in ARG composition and diversity.

As infants and young children are exposed to more antibiotics than any other age group,^19–22^ children represent an important subset of the population to understand the impact of antibiotics on their gut microbiota and ARGs. Predominant type and reason for antibiotic use vary by age and population characteristics but amoxicillin and azithromycin are typically identified as the most frequently used in young children.^23,24^ These antibiotics work through different mechanisms,^25^ but both have been associated with changes to the gut microbiome.^26,27^ Previous systematic literature reviews have explored the effect of antibiotics on the gut microbiome of children.^28,29^ Only one systematic review has assessed the gut resistome of children but this study only assessed the impact of antibiotics to the gut resistome of neonates.^29^ While understanding the impact of antibiotic exposure during the neonatal period is critical, the type, dose, duration, and indication for these antibiotic exposures is not representative of children beyond this period.

The objective of this systematic review was to assess how early childhood antibiotic exposure affects the composition and diversity of the gut resistome. In particular, we aimed to identify what is currently known about the association and what gaps persist in the literature. With the growing burden of AMR, this information can provide guidance on how to consider the resistome in antibiotic stewardship practices.

## Methods

### Protocol and registration

The protocol for this review was registered in advance to PROSPERO [CRD42021293328]. Any amendments to the protocol were noted in the **Supplement**. We used the Preferred Reporting Items for Systematic Reviews and Meta-Analyses (PRISMA) 2020 guidelines for reporting this review (see **Supplement**).^30,31^

### Eligibility criteria

Our eligibility criteria was based on our pre-specified Population, Intervention, Comparator, Outcome (PICO) framework.^31^ Specifically, we only included reports that focused on children under 5 years, had clear indication of direct systemic antibiotic exposure to the child, and included children that had a comparator or control group of children that had fewer (or zero) instances of antibiotic exposure. To ensure that our outcomes of interest could have been measured in the study, we only included studies that used non-culturable approaches to study antimicrobial resistance (e.g., whole metagenomic sequencing or qPCR). Additionally, we wanted the studies to truly be defined as resistome-wide studies so we required at least 10 ARGs to be profiled and excluded studies that only profiled phenotypic resistance in isolates. While the definition of the human gut resistome is fluid, a recent systematic review identified the minimum number of ARGs in a resistome study as 10.^17^ Reports with insufficient data on the exposure and outcomes of interest were excluded. Reports that did not directly report on the exposure and outcomes of interest but had sufficient individual-level metadata to assess both were included with re-analyzed data. We only considered randomized controlled trials and observational studies that (1) could clearly demonstrate that the exposure preceded the measurement of the resistome and (2) included a comparator group.

### Information sources and search strategy

We searched for records in PubMed (MEDLINE), Scopus, Web of Science, and the Cochrane Central Register of Controlled Trials. Only records that were available on or after January 1, 2000 were included based on related systematic reviews using this cutoff.^18,32^ Likewise, “resistome” was not used in scientific literature before 2006,^33^ so, to reduce the number of studies only assessing a subset of ARGs in the resistome but not exclude resistome-type studies published before 2006, the cutoff of 2000 was used as a conservative restriction. Only papers written in English were included due to resource limitations. In addition to the general search, RML manually reviewed references of included reports for additional research articles to include. One research article was added per suggestion during peer review.

The search strategy for each database was drafted by RML with consultation and peer review by librarians from the Dartmouth Biomedical Libraries (see **Supplement**). Our search strategy included a combination of our population (e.g., infants and children), outcome (e.g., resistance and resistome), and study system (e.g., gut and stool). Since our exposure of interest (antibiotics) is frequently intertwined with keywords used for our outcome, we chose a more conservative search strategy that did not directly include a search of antibiotics.

### Data extraction and risk of bias assessment

RML conducted the search of all databases on November 15, 2021 and extracted the information to Zotero to remove duplicates and records with any retraction notices.^34^ Entries were uploaded to Rayyan to manage data and remove any further duplicates.^35^ Rayyan is a web-based tool that enables independent screening and decision-making on record inclusion or exclusion. Two independent reviewers (RML and either DBK, JL, or HCW) completed all rounds of screening and eligibility assessment. Titles and abstracts were screened in Rayyan. Full-text of articles eligible for inclusion were uploaded to shared Zotero groups and independently reviewed for inclusion in Rayyan.

Data from all research articles were extracted separately by two independent reviewers (RML and either DBK, JL, or HCW) using a piloted standardized template (see **Supplement**) derived from The Cochrane Collaboration.^36^ Data extracted included general information on study design, total number of participants, ages of participants at the exposure and outcome measurement, setting, details on the exposure and outcome measurement, and covariate information. Information related to the bias assessment including method of recruitment and inclusion/exclusion criteria were also captured. Reports (e.g., abstracts, commentaries, and clinical trial information) from included studies were assessed in combination with research articles.

Risk of bias assessments were completed by RML and a second reviewer (DBK, JL, or HCW) independently in conjunction with the data extraction. Randomized controlled trial and observational study potential bias were assessed using different tools. Randomized controlled trials, including cluster-randomized trials, utilized risk-of-bias (RoB 2) to assess potential study-level bias due to randomization, deviation from intended interventions, missing outcome data, measurement of the outcome, and selective reporting of results.^37^ Observational studies were assessed using the Risk of Bias in Non-randomized Studies – of Interventions (ROBINS-I) tool, which assesses possible study-level bias due to confounding, selection bias, intervention classification, deviations from intended intervention usage, missing data, measurement of the outcome, and selective reporting of results.^38^ Some amendments to the ROBINS-I criteria were made to better capture biases inherent in resistome studies (see **Supplement**). Google Forms derived from the original bias assessment templates for each tool were utilized. After reviewing all articles, a joint meeting among RML, DBK, JL, and HCW was used to confirm relative bias assessment levels across both observational studies and randomized controlled trials. The *robvis* web platform was used to create stop-light figures.^39^

For studies that had multiple research articles, data extraction, and risk of bias assessment were completed separately for each research article. If studies had multiple reports of the same outcomes from different time points before 5 years of life, all outcomes were reported in the Tables. Any discrepancies or disagreements were discussed between RML and the second reviewing author. In situations where no decision was reached, disagreements were resolved by AGH. None of the reviewers were blinded to the journal titles, study authors, or author affiliations. All data extraction forms, bias assessments, instructions, and additional comments were available on the Google Drive shared with all authors.

**Table 1.** Descriptive overview of studies identified.

**Study ID**	**Aim**	**Study design**	**Country**	**Population description**	Number of eligible participants*	**Age of participants at exposure measurement**	**Antibiotic exposed participants; No.**	**Antibiotic unexposed partic- ipants; No.**	**Reason for antibiotic exposures**	**Administration of antibiotic exposures**	**Reporting of antibiotic exposures**	**Comments**
**Randomized controlled trials**												
ARMCA Study^52,53,54^	Evaluate the effect of 3 different antibiotics vs. placebo on the gut resistome of children under 5	Randomized controlled trial	Burkina Faso	Children from 2 rural comm- unities in Nouna District	120	6-59 months of age	Amoxicillin, azithromycin, or cotrim oxazole exposed to 5 days of treatment; 31 in each group randomized; 29, 31, 30 respectively with samples collected	Placebo given for 5 days of treatment; 31 rando- mized, 30 with samples collected	Prophylactic use to prevent morbidity and mortality	Pediatric oral suspension	Directly observed	
D’Souza 2020^40,55^	Analyze differences in microbiome charact eristics among HIV exposed, uninfected infants by cotrimoxazole exposure	Randomized controlled trial	South Africa	HIV-exposed, uninfected infants	63	6 weeks to 6 months of age	Cotrimoxazole exposed once daily during study period; 34	Cotrimoxazole unexposed; 29	Prophylactic use to prevent HIV	Oral	Self-report by mothers at each study visit^56^	
MORDOR Study^41,42,57,58^	Assess the effects of bi-annual mass azithromycin treatments on gut resistome of preschool children	Cluster-rando mized trial	Niger	Randomly sampled preschool children from villages in Loga and Boboye depart ments	30 communities	1-59 months of age	Mass azithromycin admini stration biannually; 15 villages	Mass placebo admini stration biannually; 15 villages	Reduce mortality	Oral	Directly observed	Outcome measurements taken at: Baseline^41,42,57^ 24 months^41,57,58^ 36 months^41,42^ 48 months^41,42^, 60 months^41^
**Observational studies**												
Bäckhed 2015^59^	Conduct a metagenomic analysis of mother-infant pairs in the first year of life to assess the impact of delivery mode and feeding	Longitudinal cohort	Sweden	Full-term vaginally delivered infants in Halland	83	Up to 1 year	Antibiotic exposure 0-4 months; 3 4-12 months; 18 Ever exposed; 21	No antibiotic exposure 0-4 months; 80 4-12 months; 65 Never exposed; 62	Variable. In infants under 4 months, mostly for bacterial infections. For antibiotics given in the 4-12 month window, mainly for upper airway and middle ear infections	Mainly orally	Medical records and national prescription registers	98 total infants were included in the study with metagenomics samples, but only the 83 vaginally delivered infants had resistome information available
Esaiassen 2018^43^	Evaluate the effect of probiotics and antibiotics on the developing gut microbiota and antibiotic resistome of preterm infants	Prospective cohort study	Norway	Premature infants from 6 NICUs	22	After first week to 4 months	Broad antibiotic exposure at 28 days; 7* Broad antibiotic exposure at 4 months; 9*	Narrow antibiotic exposure at 28 days; 15* Narrow antibiotic exposure at 4 months; 13*	Complications related to preterm birth**	Intravenous**	Medical records**	Some infants in this set were exposed to probiotics
Hourigan 2018^60^	Assess if exposure to a private or shared NICU room impacts neonatal microbiota and antibiotic resistance genes	Prospective cohort study	United States	Infants in NICU within Inova Fairfax Hospital in Falls Church, Virginia	32	Up to discharge from NICU	Antibiotic exposure ever; 20	No antibiotic use ever; 12	Complications related to preterm birth**	Intravenous**	Collected while in the NICU	Antibiotic exposure differed by NICU with infants receiving more antibiotics in the shared room (traditional) NICU compared to the private room (new) NICU
Li X. 2021^44^	Assess associations between ARGs, environmental exposures, and asthma-associated microbiome composition	Prospective cohort study	Denmark	Infants drawn from Copenhagen Prospective Studies on Asthma in Childhood 2010 birth cohort	662	Under 1 year	Antibiotic usage in first year of life; 311	No antibiotic usage in first year of life; 351	Not specified	Not specified	Measured during clinic visits and validated against registry records	
Neonatal Microbiome and Necrotizing Entero colitis Study or St Louis Neonatal Microbiome Initiative atWashington University^14,61,63^	Densely sample the gut microbiota and resistome of preterm and full-term infants exposed and unexposed to antibiotics	Longitudinal cohort	United States	Preterm infants seen in the NICU at the St. Louis Children’s Hospital and healthy term infants from the Neonatal Microbiome and Necrotizing Enterocolitis Study or St. Louis Neonatal Microbiome Initiative	6^61^ 84^63^ 58^14^	8 months^61^ Birth to hospital leave (maximum of 91 days)^63^ Up to 21 months^14^	Amoxicillin exposed for 10 days; 3^61^ Early and subsequent antibiotic exposure; 51^63^ Preterm early antibiotic exposure only or preterm early and subsequent antibiotic exposure; 9 and 32 respectively^14^	Not amoxicillin exposed for 10 days; 3^61^ Early antibiotic exposure only; 33^63^ Term antibiotic naïve infants; 17^14^	Not specified^61^ Complications due to premature birth^14,63^	Oral^61^** Predominantly intravenously^14,63^	Parental questi onnaire and verified via the infants’ medical records^61^ Not specified^14,63^	
Oulu, Finland Study^45,46^	Evaluate the combination effect of intrapartum and postnatal antibiotics on the infant gut microbiome and resistome	Prospective cohort study	Finland	Vaginally-delivered, full-term infants from Oulu University Hospital	25^45^ 20^46^	Starting within 24 hours after birth up to 7 days of life	Exposed to IAP and postnatal antibiotics; 10^45^ Exposure to IAP and postnatal antibiotics; 10 at 3-7 days; 8 at 12 months^46^	Infants not exposed to IAP or postnatal antibiotics; 15^45^ No exposure to IAP and postnatal antibiotics; 10 at 3-7 days; 8 at 12 months^46^	Clinical problems at birth reflected by low Apgar points	Intravenous	Not specified	149 infants were profiled but only a subset had resistome outcomes available
Rahman 2018^47^	Use genomes from metagenomes and machine learning to profile the resistome and accompanying gut microbes of premature infants	Longitudinal cohort	United States	Premature infants from the NICU at Magee-Women’s Hospital in Pittsburgh, Pennsylvania	107	First week to 3 months	Received antibiotics after the first week of life; 36	Received no antibiotics after the first week of life; 71	Varies but includes late-onset sepsis, NEC, or another disease	Intravenous**	Medical records**	
Reyman 2022^64,65^	Determine antibiotic regimen with least change to microbes and ARGs for suspected early-onset neonatal sepsis (sEONS) and compare to antibiotic unexposed infants	Longitudinal study	Netherlands	Infants with sEONS and age-matched infants without sEONS from multiple Dutch hospitals	225	First week of life	Exposed to amoxicillin and cefotaxime, co-amoxiclav and gentamicin, or penicillin and gentamicin; 47, 49, and 49 infants in each respective group	Not exposed to antibiotics in first week of life; 80	sEONS	Intravenous	Not specified	Study randomizes antibiotic exposure types, but not antibiotic exposure vs. no exposure (i.e., our main exposure of interest). This led to our decision to classify this study as observational for the purposde of this systematic review
Rose 2017^66^	Investigate the resistome of 11 healthy premature infants	Longitudinal cohort	United Kingdom	Healthy, preterm infants in NICU of Imperial College Healthcare Service Trust in London	11	Until initial hospital discharge (max: 43 days of life)	Exposed to course 1 antibiotics; 8 Exposed to course 2 antibiotics; 4	Not exposed to course 1 antibiotics; 3 Not exposed to course 2 antibiotics; 7	Prophylactic antibiotic treatment related to preterm birth	Intravenous**	Medical records**	
Thanert 2021^48^	Evaluate the effects of antibiotic-driven intestinal dysbiosis in gut microbiota and resistome of patients with surgically-induced short bowel syndrome (SBS) compared to preterm and term controls	Longitudinal cohort	United States	Patients with surgically-induced SBS from St. Louis Children’s Hospital	19	Up to 4 years	Current antibiotic use; 5* Antibiotic use in past month; 11*	Not current antibiotic use; 14* No antibiotic usage in past month; 8*	Not specified	Intravenous and oral	Intravenous treatments from medication histories recorded during hospita lizations and oral antibiotic treatments from clinic notes	Non-SBS controls were selected from the Neonatal Microbiome and Necrotizing Enterocolitis Study or St Louis Neonatal Microbiome Initiative at Washington University
Yassour 2016^15^	Conduct a natural history study of the gut microbiome comparing children exposed to 9 or more antibiotics versus unexposed	Longitudinal cohort	Finland	Children in the DIABIMMUNE Study from Espoo	39	Before 3 years	Received 9 or more antibiotics in first 3 years of life; 20	No antibiotic exposure in first 3 years of life; 19	Mainly to treat otitis media and respiratory infections	Systemic, orally	Reported by parents prospectively in study diary^67^	

*Eligible participants indicates children under 5 with both antibiotic exposure and resistome information **Indicates assumed value based on population characteristics (e.g., infants in NICU almost always given antibiotics intravenously)

### Main outcomes and synthesis measures

Our primary outcomes of interest were overall gene resistance load and alpha diversity of the resistome with a secondary priority of extracting ARG presence and absence data. Mean or median difference between antibiotic exposure groups for both our primary metrics (as defined by each study) were the effect measures prioritized and studies were only included in summary of findings tables if one of these metrics could be derived. Overall resistance gene load was defined as the reported relative abundance of antimicrobial resistance genes in a given sample, but exact quantification varied by study (see Table 2). Richness was prioritized over other alpha diversity metrics *post hoc* due to the availability of richness data compared to other metrics of alpha diversity in studies. Any information about these measures, certainty around the estimate, and statistical significance were included. Associations between any type of systemic antibiotic and these outcomes were considered. In instances where re-analysis of the data was required to simultaneously measure the exposure and outcome of interest, RML re-analyzed the data using the following criteria:

1. If a study had sufficient individual-level antibiotic exposure and ARG data to assess overall resistance gene load and alpha diversity by antibiotic exposure group, re-analysis was performed on these datasets using R version 3.6.0.^49^ To assess the exposure and outcomes of interest, a fixed linear regression model adjusted for the day of sample collection was used. Only one stool sample per child was included in any re-analysis. Samples collected from the window directly following the study’s classification of the antibiotic exposure window were prioritized to best reflect the direct effects of antibiotic exposure.
2. If either of these outcome variables were available by antibiotic exposure groups but had to be extracted from data tables, data from these tables was summed or combined to assess the outcome by group.
3. If either of these outcome variables were available by antibiotic exposure groups but had to be extracted from Figures, data extraction from Figures was completed using WebPlotDigitizer (https://automeris.io/WebPlotDigitizer/).

**Table 2.** Association between antibiotic exposure and overall resistance gene load.

Study ID	Antibiotic Exposure Group (N)	Comparison (N)	Age of participants at outcome measurement	ARG database used	Metric used		Unexposed or Control group	Intervention group	Difference, Percentage change	Statistical Significance	Comments
**Randomized controlled trials**											
D’Souza 2020 ^40*^	Once daily cotrimoxazole exposure during study period (N = 34)	No cotrimoxazole exposure during study period (N = 29)	4 months, 6 months	CARD		Median or mean log-transformed resistance gene abundance	Median: 4 months: 7.2 6 months: 7.1	Median: 4 months: 8.0 6 months: 8.0	Mean difference: 4 months: 0.71 (95% bootstrapped CI: 0.2–1.2) 6 months: 0.85 (0.1–1.7)	P-value: 4 months = 0.037 6 months = 0.095 (Wilcoxon test)	Median values were estimated from Figure with mean difference and p-value reported in paper
MORDOR Study ^41,42*^	Community exposed to bi-annual azithromycin (N = 14 communities)	Community exposed to bi-annual placebo (N = 15 communities)	36, 48, 60 months	MEGARes		Mean normalized abundance of resistance determinants (reads/million)	36 months: 951.4 48 months: 868.4 60 months: 699.4	36 months: 1930.6 48 months: 1661.1 60 months: 1025.3	36 months: +979.3, +102.9% 48 months: +792.6, +91.3% 60 months: + 325.9, +46.6%	Significance not calculatable for individual children	
**Observational studies**											
Esaiassen 2018 ^43*^	Broad antibiotic exposure after first week among all preterm infants (N at 28 days = 7, N at 4 months = 9)	Narrow antibiotic exposure beyond first week of life (N at 28 days = 15, N at 4 months = 13)	28 days, 4 months	CARD		Mean absolute counts/total abundance	28 days: 16.8 4 months: 23.2	28 days: 125.2 4 months: 36.3	28 days: +108.4, +645% 4 months: +13.1, +56%	Statistically significant at 28 days, not significant at 4 months	Estimates derived from table, significance reported in paper
Li X. 2021^44^	Any antibiotic exposure in first year of life (N = 311)	No antibiotic exposure in first year (N = 351)	1 year	CARD		Mean of summed ARG abundance	1223	1377	+154, +12.6%	P-value = 0.078 (Wilcoxon test)	No CI or SD given
Neonatal Microbiome and Necrotizing Enterocolitis Study or St Louis Neonatal Microbiome Initiative at Washington University ^14*^	Children with early and subsequent antibiotic exposure (N = 32)	Children born near term and antibiotic naïve (N = 17)	Up to 21 months of age	CARD		Median RPKM	1681	1971	+289.9, +17.2%	P-value < 0.05 (Wilcoxon test)	Multiple samples per child were included per Figure
Oulu, Finland Study Tapiainen 2019 ^45^^*^ Li W. 2021 ^46^^*^	Infants exposed to intrapartum and postnatal antibiotics during first week of life (N = 15)	Infants unexposed to intrapartum and postnatal antibiotics during first week of life (N = 10)	3–7 days of life	CARD		Mean AMR gene copies per sample	7560	9850	+2290,+30.3%	P-value = 0.16 (two tailed t-test)	
	(N = 8)	(N = 8)	12 months	CARD		Mean copies per million	1656.3	1757.8	+101, +6.1%	P-value = 0.93 (two tailed t-test)	
Rahman 2018 ^47**^	Antibiotic exposure after the first week (N = 36)	No antibiotic exposure after first week of life (N = 71)	Up to 86 days	Resfams		Linear regression model adjusted for day of sampling	659.3	754.8	+95.5, +14.5%	P-value = 0.07 (linear regression)	Last sample collected from infant to account for exposure window
Thanert 2021 ^48**^	Antibiotic use in the past month (N = 10)	No antibiotic use in past month (N = 9)	0–4 years	CARD and NCBI AMR database		Linear regression model adjusted for day of sampling	4234	6270.6	+2036.6, + 48.1%	P-value = 0.51 (linear regression)	Earliest sample point per child included based on exposure window

Notes: *Approximation based on values in a bar plot or box and whisker plot. **Individual-level re-analysis was conducted on the data

All re-analysis of data from individual studies was documented in **Table S1** and in the accompanying R code. Since we had a reduced level of granularity and significant heterogeneity across studies, we were not able to perform a formal meta-analysis on these data per protocol.

### Quality of evidence

Strength of evidence for each outcome was assessed based on the main components of the Grading of Recommendations Assessment, Development and Evaluation (GRADE) methodology, which assesses risk of bias, consistency of the effect, imprecision, indirectness, and other bias (see **Supplement**).^50^ Since this systematic review consisted of a mixture of randomized controlled trials and observational studies, the quality of evidence for studies started as Low per GRADE protocol but different factors were used to downgrade or upgrade the quality of evidence.^51^

## Results

### Overview of included studies

**Figure 1.**
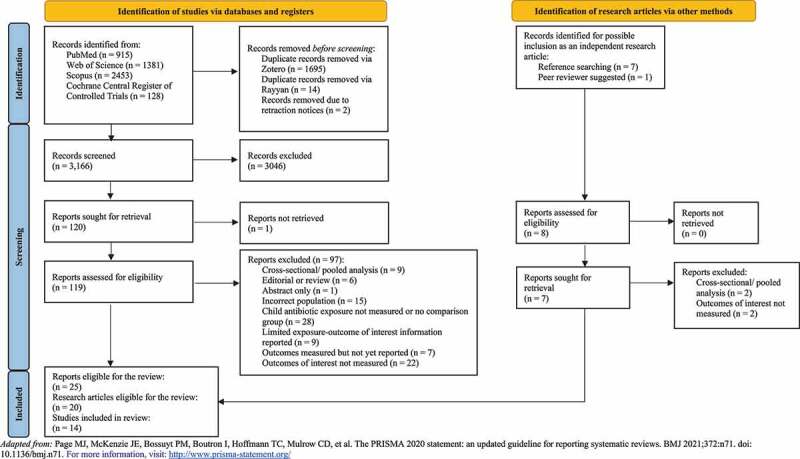
PRISMA 2020 flow diagram for new systematic reviews which included searches of databases, registers, and other sources.

From our initial search, we identified 4877 records and assessed the full-texts of 119 for eligibility (Figure 1). Additionally, we assessed seven records as independent research articles via reference searching of full-text articles that were included. A research article was added during the peer review process that was not published during our initial search. Reports were most often excluded due to: a lack of child antibiotic exposure or comparison group (n = 28), outcomes of interest not reported (n = 22) or did not meet our eligibility requirements for the participant population for this review (n = 15). Information regarding each full-text report’s inclusion or exclusion is available in **Table S2**. Ultimately, 25 reports were included in this systematic review spanning 14 independent studies (Table 1).

Of the 14 independent studies, three^40–42,52,55,57,58^ were randomized controlled trials and 11^14,15,43–48,59–67^ were observational studies. The sample sizes across studies varied widely, with many reports taking repeated samples from the same participants.^14,15,40,43,46–48,60,61,63,64^ There were several reports^15,43,45,46,48,60,61,66^ that had less than 50 participants with eligible resistome data with only a few studies capturing resistome data from over 100 participants.^41,42,44,52,57,58,64^ All the studies considered beta-lactam antibiotics except for the MORDOR Study^41,42,57,58^ which focused on antibiotic exposure to azithromycin and a study focused on evaluating the adverse effects of cotrimoxazole among HIV-exposed uninfected infants.^40,55^ All studies utilized short-read sequencing technology (e.g., Illumina HiSeq or NextSeq) or qPCR as opposed to long-read sequencing (e.g., HiFi sequencing). Different ARG databases for shotgun metagenomics were used across studies with CARD the most frequently used,^14,15,40,43–46,63^ followed, respectively, by MEGARes,^41,42,52,57,58,64^ Resfams,^14,47,63^ ARG-ANNOT,^66^ ARDB,^59^ CosmosID,^60^ PARFuMS,^61^ and the NCBI AMR database.^48^

### Overall resistance gene load

We identified eight studies^14,40–48^ with available mean or median overall resistance gene load by antibiotic exposure group data. Consistently, these studies found that there was either no difference or a statistically significant increase in overall resistance gene load between children classified as antibiotic exposed versus unexposed (Table 2). Children in the MORDOR Study were exposed to bi-annual azithromycin or placebo and followed for 60 months.^41,42,57,58^ While the MORDOR Study did not report overall resistance gene load outcomes, at both the 36 and 48 month time point, multiple resistance determinant (defined based on having a gene fraction greater than 80%)^42^ classes other than macrolides determinants were increased including beta-lactams and sulfonamides.^42^ Likewise, at these time points, all the mean resistance loads for each determinant, even if not statistically significantly different between groups, were increased in the azithromycin-exposed group.^42^ However, by 60 months there was no evidence that non-macrolide resistance determinants were different between groups.^41^ In total, these data suggest that antibiotic exposure is capable of causing collateral changes to the resistome, but these changes may only occur in a subset of children and may vary by frequency and timing of exposures. Considering these factors, we gave this outcome a Low quality of evidence value (see **Supplement**).

### Alpha diversity of ARGs

There were nine studies^14,40,44,47,48,52,59,64,66^ that assessed alpha diversity or richness by antibiotic exposure (Table 3).

**Table 3.** Association between antibiotic exposure and resistome alpha diversity.

Study ID	Antibiotic Exposure Group	Comparison	Age of participants at outcome measurement	ARG database used	Metric used	Unexposed or Control group	Intervention group	Difference, Percentage change	Significance	Comments
**Randomized controlled trials**										
ARMCA Study ^52–54^	5 day course of amoxicillin (N = 29), azithromycin (N = 31), or cotrimoxazole (N = 30)	5 day course of placebo (N = 30)	6–59 months of age	MEGARes	Mean Chao 1 richness	23.9	Amoxicillin: 42.6 azithromycin: not reported cotrimoxazole: 40.1	Amoxicillin: +18.7, +78.2% azithromycin: not reported cotrimoxazole: +16.2, +67.8%	P-value: amoxicillin = 0.02 azithromycin = 0.15 cotrimoxazole = 0.049 (t-test)	
D’Souza 2020 ^40,55^^*^	Once daily cotrimoxazole exposure during study period (N = 34)	No cotrimoxazole exposure during study period (N = 29)	4 months, 6 months	CARD	Median richness	4 months: 41.3 6 months: 40.1	4 months: 62.6 6 months: 58.9	4 months: +21.3, +51.5% 6 months: +18.8, +47.0%	P-value = 0.005 (mixed effects model estimate)	Significance value derived from mixed effects model in D’Souza 2020^40^ that adjusted for time, read count, and a random intercept for infant
**Observational studies**										
Bäckhed 2015 ^59^^*^	Antibiotics after 4 months (N = 18)	No antibiotics after 4 months (N = 65)	12 months	ARDB	Mean number of resistance types	12 months: 31.5	12 months: 29.5	12 months: -1.8, −5.6%	P-value = NS (Wilcoxon test)	Significance reported in manuscript
Li X. 2021^44^	Any antibiotic exposure in first year of life (N = 311)	No antibiotic exposure in first year (N = 351)	1 year	CARD	Mean richness	55	54	−1, −1.8%	P-value = 0.5 (one-way ANOVA)	
Neonatal Microbiome and Necrotizing Enterocolitis Study or St Louis Neonatal Microbiome Initiative at Washington University ^14^^*^	Children with early and subsequent antibiotic exposure (N = 32)	Children born near term and antibiotic naïve (N = 17)	Up to 21 months of age	CARD	Median richness	97.3	84.7	−12.6, −13.0%	P-value < 0.05 (Wilcoxon test)	Multiple samples per child were included
Rahman 2018 ^47^^**^	Antibiotic exposure after the first week of life (N = 36)	No antibiotic exposure after first week of life (N = 71)	Up to 86 days	Resfams	Linear regression model adjusted for day of life	32.3	29.6	−2.7, −8.4%	P-value = 0.09 (linear regression)	Last sample collected from infant to account for exposure window
Reyman 2022^64^	Antibiotic exposure for sEONS in first week of life (N = 147)	No antibiotic exposure for sEONS in first week of life (N = 78)	1 month	(qPCR Fluidigm primers used; some match reported organisms from the Antibiotic Resistance Database)	Median observed number (richness)	7.5	9	+1.5, +20%	P-value = 0.02 (Wilcoxon test)	AMR gene diversity did not differ by antibiotic status in cross-sectional analysis at other ages or in a temporal analysis over the first year of life
Rose 2017 ^66^^**^	Infants exposed to one or two courses (N = 9)	Infants unexposed (N = 2)	Up to 43 days of life	ARG-ANNOT	Linear regression model adjusted for day of sampling	9.2	5.9	−3.3, −35.8%	P-value = 0.45 (linear regression)	
Thanert 2021 ^48^^**^	Antibiotic use in the past month (N = 10)	No antibiotic use in past month (N = 9)	0–4 years	CARD and NCBI AMR database	Linear regression model adjusted for day of sampling	37.8	45.2	+7.4, +19.5%	P-value = 0.59 (linear regression)	Earliest sample point per child included based on exposure window

Notes: *Approximation based on values in a bar plot or box and whisker plot. **Individual-level re-analysis was conducted on the data

The two randomized controlled trials that evaluated richness identified children exposed to amoxicillin^52^ or cotrimoxazole^40,52^ both had statistically significant increases in the number of unique ARGs in antibiotic treated children compared to the unexposed or placebo group. In contrast, observational studies in populations of generally healthy infants born predominantly at term exposed to greater quantities of antibiotics had a statistically significant but only slight increase in ARG richness at 1 month,^64^ approximately the same number of resistance gene types,^44,59,64^ or a decreased richness of ARGs.^14^ While these results are discrepant, they are likely a reflection of the underlying population characteristics and microbial composition. A “central dogma” of the gut microbiome field is that participants that have a higher microbial alpha diversity or greater strain variation are better able to respond to perturbations.^16^ Since the resistome is interconnected with the gut microbiome,^14,44,68^ results for resistome alpha diversity are likely related. For instance, in Gasparrini et al.^14^ out of 54 metagenomes with high resistance loads, 41 (76%) were dominated by a single species. *Escherichia coli* was the most frequently identified dominant species, but other dominant species included *Enterococcus faecalis*, and *Klebsiella pneumoniae*. They found alpha diversity was statistically significantly lower among children with early and subsequent antibiotic exposure compared to near-term children unexposed to antibiotics. Meanwhile, Li et al.^44^ also found that *E. coli* abundance contributed to the resistome profile, but at a smaller abundance per sample. No difference in ARG richness was noted between exposed and unexposed infants. Thus, not only which microbes are present, but their relative abundance in a given population is likely to be a main driver of the heterogeneous findings.

### Studies separated into 3 groups

While there was much heterogeneity across studies included, we identified three main categories defined by study design, participants, country, and antibiotic exposure (Table 4). These groups were used to summarize our findings for outcomes of interest and identify gaps in the literature.

**Table 4.** The effect of antibiotic exposures by different study groups.

	Study design	Participants	Country	Antibiotic Exposures	Research question	Key Findings
Group 1^40–42,52,57,58^	Randomized controlled trials	Children <5 years old in communities with a high burden of infection and malnutrition	Niger,^41,42,57,58^ South Africa,^40^ Burkina Faso^52^	Macrolides (azithromycin), ^41,42,52,57,58^ sulfanilamides (cotrimoxazole),^40,52^ beta-lactams (amoxicillin)^52^	How does prophylactic antibiotic use to prevent morbidity and mortality impact the gut resistome?	Consistent evidence of increased abundance and diversity of ARGs conferring resistance to antibiotic given with some evidence suggesting increases to other ARGs.
Group 2^15,44–46,59,61,64^	Longitudinal, observational studies	Generally healthy populations of children <3 years old	Finland,^15,45,46^ Sweden,^59^ Denmark,^44^ Netherlands,^64^ USA^61^	Variety of antibiotics including: beta-lactams,^15,44–46,59,61,64^ aminoglycosides,^15,44–46,64^ sulfanilamides,^15,44^ macrolides^15,44^	How does typical antibiotic use for common childhood ailments impact gut resistome?	Studies in this category suggest that overall resistance gene load and ARG alpha diversity are not significantly impacted by antibiotics, but specific ARGs do increase after exposure to antibiotics.
Group 3^14,43,47,60,63,66^	Longitudinal, observational studies of children selected based on prematurity or NICU exposure	Predominantly children <2 years old born preterm or infants in NICU	Norway,^43^ USA,^14,60,63^ UK^66^	Greatest variety of antibiotics including: beta-lactams,^14,43,47,60,63,66^ aminoglycosides,^14,43,47,60,63^ vancomycin,^14,60,63,66^ clindamycin,^14,63^ sulfanilamides,^14,63^ macrolides^47^	How does antibiotic use during early-life hospitalization impact the gut resistome?	The microbiome is less resilient and most likely to be affected by perturbation. This leads to the highest levels of resistome heterogeneity between studies.

The studies in the first group^40–42,52,57,58^ were randomized controlled trials that had the aim of assessing whether prophylactic antibiotic exposure to prevent infection or reduce malnutrition affects the gut resistome. Two of the studies were focused at the individual level,^40,52,55^ while the unit of analysis for the MORDOR Study was at the *grappe* or village level.^41,42,57,58^ Across these studies, there was evidence suggesting that azithromycin or cotrimoxazole exposure was associated with increased prevalence of associated ARGs. In the ARMCA Study,^52–54^ macrolide ARG prevalence was 87.1% in children exposed to azithromycin compared to 33.3% in the control group. Similarly, macrolide ARG prevalence was 68.0% in communities exposed to azithromycin compared to 46.7% in the placebo-controlled communities at 24 months in the MORDOR Study.^57^ For cotrimoxazole, in the ARMCA Study,^52–54^ the risk ratios comparing exposed and unexposed children for both sulfonamide and trimethoprim resistance genes were significant [sulfonamide: 8.83 (95% CI: 1.01–77.0) and trimethoprim: 3.29 (95% CI: 1.08 to 9.95)]. Similarly, *dfr* and *sul* resistance gene richness for exposed infants was greater than in unexposed infants (mixed effects linear regression *p*-value = 0.016). The studies also support the notion that antibiotics affect ARGs beyond those conferred by the antibiotic but likely not to the same extent. Specifically, in the MORDOR Study, they found that beta-lactam resistance gene determinants were increased by a factor difference of 2.13 (95% CI: 1.33–4.02) and 1.98 (95% CI: 1.10–4.57) at 36 and 48 months^42^ respectively but did not differ by exposure group at 60 months.^41^ Neither the prevalence of these resistance determinants nor other non-macrolide determinants differed at 24 months.^57,58^ Similarly, none of the antibiotics assessed in the ARMCA Study^52–54^ resulted in a prevalence difference in beta-lactam resistance genes but both amoxicillin and azithromycin exposure were associated with increased prevalence of sulfonamide resistance genes [risk ratio for amoxicillin: 15.3 (95% CI: 1.80–129.1) and azithromycin: 16.0 (95% CI 1.91–133.5)] in children 6–59 months of age.

The second group^15,44–46,59,61,64^ assessed the effects of antibiotic exposures primarily for common infections, such as clinical concerns requiring antibiotics in the first week of life,^45,46,64^ respiratory illnesses,^15,59^ and otitis media^15,59^ afflicting children under 3. All but one^61^ of the reports were jointly interested in the impacts of multiple early life factors in addition to direct antibiotic exposure including delivery method,^15,44,59,64^ maternal antibiotic exposures,^44–46,59,64^ the child’s diet,^15,44,59,64^ and other environmental exposures.^44,64^ Multiple studies also profiled the maternal microbiome with the goal of understanding if antibiotic exposure modifies vertical transfer of microbes or ARGs.^45,46,59,61^ All studies included infants up to 1 year of age and only one study had information on children 1–3 years of age.^15^ Compared to studies from the other groups, we found evidence that the resistome of children in this group is less sensitive to the effects of antibiotic exposure. These main findings are discussed in Yassour et al.^15^ and Moore et al.^61^ and is evidenced by the null effects of antibiotic exposure on overall resistance gene load and alpha diversity as noted in other studies.^44–46,59^ However, while overall resistome outcomes may only be moderately impacted by antibiotic exposure, antibiotic exposure can still impact individual ARGs via alterations to microbial composition. As identified in Yassour et al.,^15^ certain ARGs on chromosomes may peak in abundance directly after antibiotic exposure and then decline, while some ARGs on mobile genetic elements persist long after antibiotic exposure. The authors found that the abundance changes of ARGs on chromosomes was correlated to the abundance of species, such as *E. coli, Klebsiella pneumoniae*, and *Ruminococcus gnavus*. This agrees with Li X. et al.^44^ which found *E. coli* to describe the resistome pattern and the larger literature^68–70^ suggesting *E. coli* abundance has a major influence on ARG abundance in infants from generally healthy children cohorts.

The last group of studies^14,43,47,60,62,63,66^ had the broad aim of understanding how early life antibiotic exposures impact the gut resistome of children born prematurely or with extended exposure to the Neonatal Intensive Care Unit (NICU). Only Gasparrini et al.^14^ evaluated children beyond 4 months with the focus primarily on antibiotic exposures occurring during early life hospitalization. This was also the only study to compare the resistome of preterm infants to near-term infants.^14^ Hourigan et al.^60^ attempted to separate the effects of hospitalization and antibiotic exposure, but identified that both components impacted the abundance of ARGs. Compared to studies in Group 2, the children in Group 3 studies have resistome compositions more sensitive to antibiotic exposure. The overall resistance gene load was increased^14,43,47^ in children exposed to any or multiple antibiotic exposures compared to unexposed infants with specific ARGs differentially abundant in antibiotic-exposed children.^60,62,63^ Interestingly, studies in this set looking at richness^14,47,66^ found an inverse association between antibiotic exposure and ARG richness. Similarly to studies in other groups, changes to gut microbiota impacted the resistome^14,47,63,66^ with multiple studies noting that species dominance (i.e., the species comprises >50% of the sample) was an important factor in resistome composition.^14,63,66^

In addition to the three main groups of studies, Thanert et al.^48^ was focused on children with surgically induced short bowel syndrome (SBS) making it distinct from other studies. Neither overall resistance gene load or alpha diversity of ARGs differed among children exposed or unexposed to antibiotics in the previous month in this study.

### Potential sources of bias in included studies

**Figure 2.**
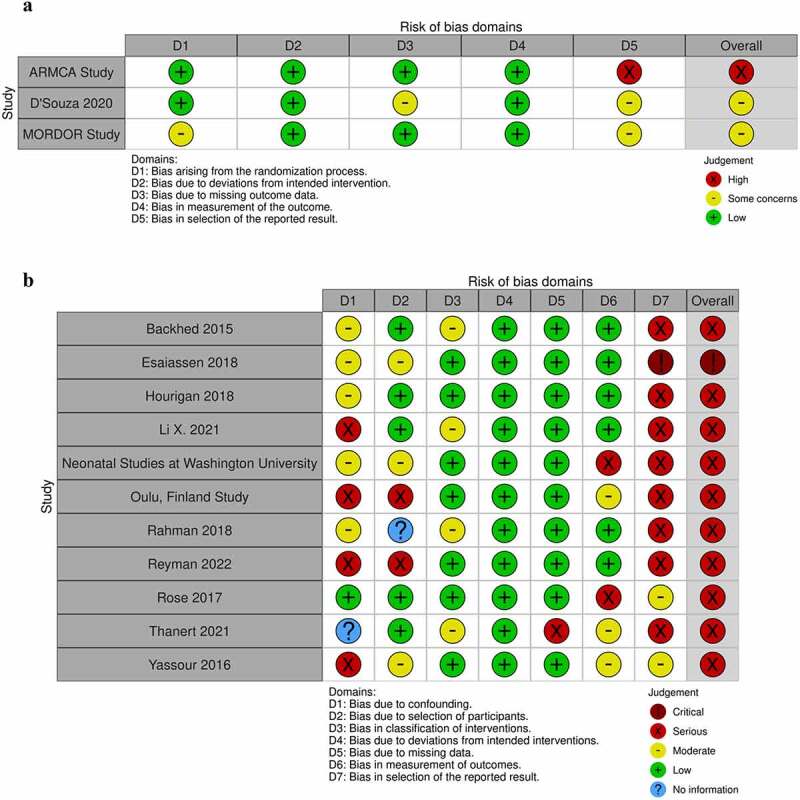
Assessment of potential bias sources for included a) randomized controlled trials and b) observational studies. Randomized controlled trials were assessed using the RoB 2 tool while observational studies were assessed using ROBINS-I.

Our bias assessment revealed some potential sources of bias from all included studies related to their assessment of antibiotic exposure and the resistome (Figure 2 and **Table S3**). The predominant potential bias identified was bias due to the selective reporting of results, which was heavily affected by lack of publicly available protocols or statistical analysis plans for observational studies. While we amended the ROBINS-I guidelines for observational studies to account for particular challenges facing resistome studies including widespread unmeasured intra- and inter-individual variation (see **Supplement**), among observational studies, most studies presented potential for unmeasured confounding of the association between antibiotic exposures and the resistome. Likewise, while all studies measured the same ARGs across antibiotic exposed and unexposed children, some studies took samples at a different time,^45,46,66^ or more frequently,^14,15,48,63^ in antibiotic exposed children.

## Discussion

While antibiotic exposures are known to have unintended side effects, limited prior research has assessed the impact of antibiotics on the gut resistome. As children are frequently exposed to antibiotics and childhood represents a sensitive window of microbiome development, it is necessary for researchers to study the effects of antibiotic exposure on the gut resistome to create better antibiotic stewardship guidelines. The objectives of this systematic review were to highlight the known impact of antibiotics on the gut resistome of young children, compare heterogeneity across study findings, identify current gaps in the field, and reveal potential bias across studies.

We found evidence that antibiotics frequently but not always impact the gut resistome of young children. These results are likely affected by the differences in the half-lives, dose, duration, and mechanism of action of the antibiotic exposure(s).^71^ Studies that assessed the effect of antibiotics on the overall resistance gene load found either no association^43,44,46,47^ or a positive association.^14,40,43,45^ Results for richness or alpha diversity were inconsistent with studies identifying that antibiotics led to a decreased^14^ or increased^40,52,64^ alpha diversity of ARGs while other studies identified no statistically significant association^44,47,59,64,66^ with some variation by time points measured. These results are similar to a systematic review that found antibiotics impact microbial diversity inconsistently depending on the antibiotic exposure and population.^28^ While this systematic review did not prioritize abundance changes of individual or classes of ARGs due to heterogeneity in reference databases and metrics used to assess individual ARGs, future research is needed to establish how these overall resistome changes translate to specific antibiotic-resistant organisms.

In this systematic literature review, we identified three main groups of studies. These groups represent significantly different populations with distinct disease burdens and indications for antibiotic exposures. Results for our main outcomes differed by study group, but it’s worthwhile to mention that likely the strongest evidence that antibiotics impact the resistome come from the Group 1 studies due to the combination of randomization and direct observation of antibiotic exposures. Regardless, in crafting resistome-conscious antibiotic stewardship practices, a focused assessment of children from each population will likely offer the most opportunities for impact. This is an especially important consideration for studies that did not assess prophylactic antibiotic exposure that may be affected by confounding by indication (i.e., the effect of the antibiotic vs. the effect of the infection).^72,73^ Beyond these study groupings, there is a dearth of knowledge available to evaluate if antibiotics impact the gut resistome in young children. Additional studies assessing associations in populations from additional georgraphies and with varying diseases or disease risks (e.g., type I diabetes, irritable bowel disease, and cystic fibrosis) may help clarify the overall effects of antibiotics to the child gut resistome.

While this systematic review was able to expose the impact of antibiotics on the gut resistome of young children, assesses potential biases in the field, and identify gaps in the literature, there were some limitations. One primary limitation we noted was the lack of consistency in reported information for both the exposure and outcomes of interest. This heterogeneity prevented formal meta-analysis of the data. Even without formal meta-analysis, we were able to identify consistent trends in the association for overall resistance gene load and different resistome alpha diversity trends by study grouping. This heterogeneity and lack of standardization in the microbiome and resistome field is an ongoing concern,^74^ but guidelines, such as the STORMS checklist^75^ could be beneficial. An additional limitation of this study was that we only used publicly available data. This decision was made as we felt that our goals were not oriented to quantify an exact metric for our outcomes. However, this did have an impact on our bias assessment. In particular, based on ROBINS-I criteria, many observational studies received a “Serious” categorization for potential bias due to selective reporting as many had no publicly available protocol. While there is certainly potential for much bias based on not having an advanced protocol, it’s worthwhile to emphasize that potential does not necessary equate to actualized bias. An individual-level meta-analysis of antibiotic and resistome data pulled from a database of studies that is able to standardize and normalize metrics would provide a better quantitative measurement with less potential bias.^76^ Another limitation of this systematic review is that it did not focus on the abundance of individual ARGs nor ARG classes. As antibiotic exposure is likely to have heterogeneous effects on different ARGs, not all ARGs confer the same risk of leading to an antibiotic-resistant infection,^7^ and there are variable definitions of the number of ARGs that define the resistome,^17^ additional systematic reviews could help disentangle the impact of antibiotics on specific components of the resistome of young children. Lastly, we did not conduct a formal publication bias analysis per protocol, but there is evidence that this could be a concern. In particular, nine studies were excluded from our systematic review due to limited information tying the exposure and outcome of interest for this systematic review together.^68,77–84^ For these studies, we also could not identify publicly available individual-level data for re-analysis. Some studies likely did not focus on the association between antibiotic exposure and resistome outcomes due to a small sample size among antibiotic exposed children^68,82,84^ and thus would not have been powered to conduct a significant analysis. Without knowledge of protocols set in advance of data analysis, it’s unclear if other studies focused on antibiotic exposure due to a lack of association identified or because the authors prioritized assessing other exposure-outcome associations. Additional studies may clarify the associations discussed in this review, as we identified five clinical trials^85–89^ that are planning to incorporate information on the exposure and outcome of interest.

## Conclusion

This systematic review found clear evidence that antibiotics impact the gut resistome of young children, but that additional studies are needed to evaluate the duration and extent. Potential bias across these studies is high with selective reporting of results and confounding major concerns that contributed to low confidence in the quality of quantitative evidence in this review. Additional studies in the field could help identify ideal antibiotic stewardship practices that consider the heterogeneity of the resistome in every population.

## Supplementary Material

Supplemental Material

## Data Availability

All data used in this manuscript is publicly available. The R Markdown script is available on GitHub (https://github.com/hoenlab/Antibiotics-on-Gut-Resistome-of-Young-Children-Systematic-Review.git). A template data extraction form is available in the **Supplement** and data extraction forms for each study are available upon request to the corresponding author.
