# Supplementary material for: The association between early life antibiotic exposure and the gut resistome of young children: a systematic review
Source: Gut Microbes. 2022 Oct 26;14(1):2120743. doi: 10.1080/19490976.2022.2120743 (PMC9621065; doi:10.1080/19490976.2022.2120743)
Supplement: Supplemental Material [file KGMI_A_2120743_SM4141.zip › Supplement_Antibiotics_Resistome_SystematicReview_July28 .docx]

**Title:** The association between early life antibiotic exposure and the gut resistome of young children: a systematic review

**Author line:** Rebecca M. Lebeaux, Despina B. Karalis, Jihyun Lee, Hanna C. Whitehouse, Juliette C. Madan, Margaret R. Karagas, Anne G. Hoen

**Table of Contents**

**Amendments to the protocol**…………………………………………..…………………....2

**PRISMA 2020 checklist**……………………………………………………………………….4

**Search strategy results**………………………………………………………………….……7

**Search strategy peer review** ………………………………………………………..……..13

**PRESS peer review checklist**…………………………………………………………..…..19

**Example data extraction form** …………………………………………………..………...21

**Amendments to ROBINS-I criteria**………………..……………………………………….31

**GRADE: Quality of evidence for resistome outcomes**….………………..……………35

**Amendments to the protocol**

| **Protocol Description Section** | **Amendment** | **Rationale** |
| --- | --- | --- |
| Risk of bias (quality) assessment | The *robvis* software web interface was used instead of RevMan software. | The stop-light figures were simpler to create using the *robvis* web platform compared to the RevMan software for ROBINS-I, so a decision was made to use *robvis* software for both. |
| Measures of effect | Mean or median difference between antibiotic-exposed and unexposed children was considered. | Due to the presentation of the center of data in many resistome studies, we decided to consider the median difference between antibiotic groups in addition to the mean as a main measure of effect. |
| Data extraction | Initial data extraction was conducted by two reviewers. After reviewing all included studies, studies were re-analyzed by RML as necessary to assess antibiotic exposure and outcomes (overall resistance gene load and alpha diversity) of interest. (Additional details on the re-analysis are below.) | Many studies contained information on the exposure and outcomes of interest but did not present them based on our preferred measures of effect, requiring re-analysis. |
| Strategy for data synthesis | If a study had sufficient metadata and ARG data at the individual-level, this data was prioritized. A fixed regression model with the exposure and outcome adjusting for day of life was utilized. Antibiotic exposure/no exposure was prioritized in the window directly following the study’s classification of the antibiotic exposure window. Studies without sufficient individual-level data but with exposure/outcome information data from Figures or Tables were incorporated. Data extraction from Figures was completed using WebPlotDigitizer (<https://automeris.io/WebPlotDigitizer/>). | Individual-level data was the most specific way to pull information from studies and was thus prioritized. We prioritized antibiotic exposure/no exposure and the closest window or sample to each study’s antibiotic exposure window. Only one sample per child was included. Figure data extraction using a tool provided better granularity of data that estimating manually. |
| Strategy for data synthesis | We did not contact authors of studies. | The authors decided that only using publicly available data was best given that extensive information would need to be collected from every study and estimates could be pulled for each study. Future studies that are able to standardize data at the individual-level would be appropriate to analyze this data. |
| Risk of bias (quality) assessment | The strength of evidence for each outcome was assessed based on the main components of the Grading of  Recommendations Assessment, Development and Evaluation (GRADE) methodology  which assesses risk of bias, consistency of the effect, imprecision, indirectness, and  other bias [The GRADE approach is reproducible in assessing the quality of  evidence of quantitative evidence synthesis; Mustafa et al. J Clin Epidemiol. 2013;66]. | We were unable to conduct a formal meta-analysis on the data and thus could not formally assess GRADE outcomes. A chart with our rationale is provided at the end of this supplement. |

**PRISMA 2020 Checklist**

| **TITLE** | | |  |
| --- | --- | --- | --- |
| Title | 1 | Identify the report as a systematic review. | Title, pg 1 |
| **ABSTRACT** | | |  |
| Abstract | 2 | See the PRISMA 2020 for Abstracts checklist. | Abstract, pg 2-3 |
| **INTRODUCTION** | | |  |
| Rationale | 3 | Describe the rationale for the review in the context of existing knowledge. | Introduction, pg 4-5 |
| Objectives | 4 | Provide an explicit statement of the objective(s) or question(s) the review addresses. | Introduction, pg 5 |
| **METHODS** | | |  |
| Eligibility criteria | 5 | Specify the inclusion and exclusion criteria for the review and how studies were grouped for the syntheses. | Eligibility criteria (Methods), pg 6-7 |
| Information sources | 6 | Specify all databases, registers, websites, organisations, reference lists and other sources searched or consulted to identify studies. Specify the date when each source was last searched or consulted. | Information sources and search strategy, Data extraction and risk of bias assessment (Methods), pg 7 |
| Search strategy | 7 | Present the full search strategies for all databases, registers and websites, including any filters and limits used. | Search strategy results (Supplement), pg 7-12 |
| Selection process | 8 | Specify the methods used to decide whether a study met the inclusion criteria of the review, including how many reviewers screened each record and each report retrieved, whether they worked independently, and if applicable, details of automation tools used in the process. | Data extraction and risk of bias assessment (Methods), pg 7-9 |
| Data collection process | 9 | Specify the methods used to collect data from reports, including how many reviewers collected data from each report, whether they worked independently, any processes for obtaining or confirming data from study investigators, and if applicable, details of automation tools used in the process. | Data extraction and risk of bias assessment (Methods), pg 7-9 |
| Data items | 10a | List and define all outcomes for which data were sought. Specify whether all results that were compatible with each outcome domain in each study were sought (e.g. for all measures, time points, analyses), and if not, the methods used to decide which results to collect. | Main outcomes and synthesis measures (Methods), pg 9-11 |
|  | 10b | List and define all other variables for which data were sought (e.g. participant and intervention characteristics, funding sources). Describe any assumptions made about any missing or unclear information. | Data extraction and risk of bias assessment (Methods), pg 8. Example data extraction form (Supplement), pg 21-30 |
| Study risk of bias assessment | 11 | Specify the methods used to assess risk of bias in the included studies, including details of the tool(s) used, how many reviewers assessed each study and whether they worked independently, and if applicable, details of automation tools used in the process. | Data extraction and risk of bias assessment (Methods), pg 8-9 |
| Effect measures | 12 | Specify for each outcome the effect measure(s) (e.g. risk ratio, mean difference) used in the synthesis or presentation of results. | Main outcomes and synthesis measures (Methods), pg 9-10 |
| Synthesis methods | 13a | Describe the processes used to decide which studies were eligible for each synthesis (e.g. tabulating the study intervention characteristics and comparing against the planned groups for each synthesis (item #5)). | Main outcomes and synthesis measures (Methods), pg 9-10 |
|  | 13b | Describe any methods required to prepare the data for presentation or synthesis, such as handling of missing summary statistics, or data conversions. | Main outcomes and synthesis measures (Methods), pg 9-10 |
|  | 13c | Describe any methods used to tabulate or visually display results of individual studies and syntheses. | Main outcomes and synthesis measures (Methods), pg 9-10 |
|  | 13d | Describe any methods used to synthesize results and provide a rationale for the choice(s). If meta-analysis was performed, describe the model(s), method(s) to identify the presence and extent of statistical heterogeneity, and software package(s) used. | Main outcomes and synthesis measures (Methods), pg 9-10. |
|  | 13e | Describe any methods used to explore possible causes of heterogeneity among study results (e.g. subgroup analysis, meta-regression). | Studies separated in 3 groups (Results), pg 24-26. |
|  | 13f | Describe any sensitivity analyses conducted to assess robustness of the synthesized results. | N/A |
| Reporting bias assessment | 14 | Describe any methods used to assess risk of bias due to missing results in a synthesis (arising from reporting biases). | Amendments to ROBINS-I criteria (Supplement), pg 31-34 |
| Certainty assessment | 15 | Describe any methods used to assess certainty (or confidence) in the body of evidence for an outcome. | Quality of evidence (Methods), pg 11 |
| **RESULTS** | | |  |
| Study selection | 16a | Describe the results of the search and selection process, from the number of records identified in the search to the number of studies included in the review, ideally using a flow diagram. | Figure 1, pg 13 |
|  | 16b | Cite studies that might appear to meet the inclusion criteria, but which were excluded, and explain why they were excluded. | Overview of included studies, (Results), pg 12 |
| Study characteristics | 17 | Cite each included study and present its characteristics. | Table 1, pg 14-17 |
| Risk of bias in studies | 18 | Present assessments of risk of bias for each included study. | Figure 2 pg 29. Table S3 |
| Results of individual studies | 19 | For all outcomes, present, for each study: (a) summary statistics for each group (where appropriate) and (b) an effect estimate and its precision (e.g. confidence/credible interval), ideally using structured tables or plots. | Table 2 and 3, pg 20-23 |
| Results of syntheses | 20a | For each synthesis, briefly summarise the characteristics and risk of bias among contributing studies. | Overall gene resistance load and alpha diversity of ARGs (Results), pg 18 and 19 |
|  | 20b | Present results of all statistical syntheses conducted. If meta-analysis was done, present for each the summary estimate and its precision (e.g. confidence/credible interval) and measures of statistical heterogeneity. If comparing groups, describe the direction of the effect. | Table 2 and 3, pg 20-23. Overall gene resistance load and alpha diversity of ARGs (Results), pg 18 and 19 |
|  | 20c | Present results of all investigations of possible causes of heterogeneity among study results. | Table 4, pg 28. Studies separated into 3 groups (Results), pg 24-26 |
|  | 20d | Present results of all sensitivity analyses conducted to assess the robustness of the synthesized results. | N/A |
| Reporting biases | 21 | Present assessments of risk of bias due to missing results (arising from reporting biases) for each synthesis assessed. | N/A |
| Certainty of evidence | 22 | Present assessments of certainty (or confidence) in the body of evidence for each outcome assessed. | Quality of evidence (Supplement), pg 35 |
| **DISCUSSION** | | |  |
| Discussion | 23a | Provide a general interpretation of the results in the context of other evidence. | Discussion, pg 30 |
|  | 23b | Discuss any limitations of the evidence included in the review. | Discussion, pg 31-33 |
|  | 23c | Discuss any limitations of the review processes used. | Discussion, pg 30-32 |
|  | 23d | Discuss implications of the results for practice, policy, and future research. | Conclusion, pg 34 |
| **OTHER INFORMATION** | | |  |
| Registration and protocol | 24a | Provide registration information for the review, including register name and registration number, or state that the review was not registered. | Protocol and registration (Methods), pg 6 |
|  | 24b | Indicate where the review protocol can be accessed, or state that a protocol was not prepared. | Protocol and registration (Methods), pg 6 |
|  | 24c | Describe and explain any amendments to information provided at registration or in the protocol. | Protocol and registration (Methods), pg 6. Amendments to the protocol (Supplement), pg 2-3. |
| Support | 25 | Describe sources of financial or non-financial support for the review, and the role of the funders or sponsors in the review. | Funding details, pg 46 |
| Competing interests | 26 | Declare any competing interests of review authors. | Disclosure statement, pg 46 |
| Availability of data, code and other materials | 27 | Report which of the following are publicly available and where they can be found: template data collection forms; data extracted from included studies; data used for all analyses; analytic code; any other materials used in the review. | Data availability statement, pg 46 |

*From:*  Page MJ, McKenzie JE, Bossuyt PM, Boutron I, Hoffmann TC, Mulrow CD, et al. The PRISMA 2020 statement: an updated guideline for reporting systematic reviews. BMJ 2021;372:n71. doi: 10.1136/bmj.n71

For more information, visit: <http://www.prisma-statement.org/>

**Search strategy results**

**Concept Map Overview:**

| **Concept** | **Keywords (tiab)** | **MeSH (mesh)** |
| --- | --- | --- |
| Antibiotic resistance genes | Antibiotic resistan*, antimicrobial resistan*, antibacterial resistan*, anti-bacterial resistan*, anti-microbial resistan*, resistome*, mobile genetic element*, resistance gene* | Drug Resistance, Bacterial |
| Child | Infant*, pedi*, paed*, child*, neonat*, newborn*, baby, babies, toddler*, preschool, pre-school | Infant; Child, Preschool |
| Gut | Stool, fecal, faecal, gut, feces, faeces, intestin*, gastro* | Gastrointestinal Microbiome; Gastrointestinal Tract; Feces |

**Web of Science**

Date Accessed November 15, 2021

Publication date: 1/1/2000 – 1/1/3000

|  | **Topic Search (includes title, abstract, and keyword)** | **Results returned** | **Query Link** |
| --- | --- | --- | --- |
| 1 | TS=(“antibiotic resistan*” OR “antimicrobial resistan*” OR “antibacterial resistan*” OR “anti-bacterial resistan*” OR “anti-microbial resistan*” OR resistome* OR “mobile genetic element*” OR “resistance gene*”) and Articles or Proceedings Papers (Document Types) and English (Languages) | 96,558 | [Link](https://www.webofscience.com/wos/woscc/summary/cade0e26-eff4-4ea3-9f08-e858ea97c787-1346e72d/relevance/1) |
| 2 | TS=(infant* OR pedi* OR paed* OR child* OR neonat* OR newborn* OR baby OR babies OR toddler* OR preschool OR pre-school) and Articles or Proceedings Papers (Document Types) and English (Languages) | 1,567,128 | [Link](https://www.webofscience.com/wos/woscc/summary/40f75bc0-83cb-4d48-aa40-0300304eb070-1346ee3b/relevance/1) |
| 3 | TS=(stool OR fecal OR faecal OR gut OR feces OR faeces OR intestin* OR gastro*) and Articles or Proceedings Papers (Document Types) and English (Languages) | 590,473 | [Link](https://www.webofscience.com/wos/woscc/summary/4f87d5d4-6674-4588-992c-02522612227b-1346f356/relevance/1) |
| 4 | #1 AND #2 AND #3 | 1,381 | [Link](https://www.webofscience.com/wos/woscc/summary/daec118a-46dc-476c-85d0-3e47167e6237-1346fee0/relevance/1) |

**Scopus**

Date Accessed: November 15, 2021

|  | **Search** | **Results returned** |
| --- | --- | --- |
| 1 | TITLE-ABS-KEY ( “antibiotic resistan*” OR “antimicrobial resistan*” OR “antibacterial resistan*” OR “anti-bacterial resistan*” OR “anti-microbial resistan*” OR resistome* OR “mobile genetic element*” OR “resistance gene*” ) AND PUBYEAR > 1999 AND ( LIMIT-TO ( DOCTYPE , “ar” ) OR LIMIT-TO ( DOCTYPE , “cp” ) ) AND ( LIMIT-TO ( LANGUAGE , “English” ) ) | 137,127 |
| 2 | TITLE-ABS-KEY ( infant* OR pedi* OR paed* OR child* OR neonat* OR newborn* OR baby OR babies OR toddler* OR preschool OR pre-school ) AND PUBYEAR > 1999 AND ( LIMIT-TO ( DOCTYPE , “ar” ) OR LIMIT-TO ( DOCTYPE , “cp” ) ) AND ( LIMIT-TO ( LANGUAGE , “English” ) ) | 2,020,066 |
| 3 | TITLE-ABS-KEY ( stool OR fecal OR faecal OR gut OR feces OR faeces OR ntestine* OR gastro* ) AND PUBYEAR > 1999 AND ( LIMIT-TO ( DOCTYPE , “ar” ) OR LIMIT-TO ( DOCTYPE , “cp” ) ) AND ( LIMIT-TO ( LANGUAGE , “English” ) ) | 771,473 |
| 4 | #1 AND #2 AND #3 | 2,453 |

**PubMed**

Date Accessed: November 15, 2021

|  | **Search** | **Number Results Returned** |
| --- | --- | --- |
| 1 | ("antibiotic resistan*"[Title/Abstract] OR "antimicrobial resistan*"[Title/Abstract] OR "antibacterial resistan*"[Title/Abstract] OR "anti-bacterial resistan*"[Title/Abstract] OR "anti-microbial resistan*" [Title/Abstract] OR resistome* [Title/Abstract] OR "mobile genetic element*"[Title/Abstract] OR "resistance gene*"[Title/Abstract]) NOT (Animals[Mesh] NOT (Animals[Mesh] AND Humans[Mesh])) AND (2000/1/1:3000/12/12[pdat]) AND (english[Filter]) | 36,550 |
| 2 | "drug resistance, bacterial"[MeSH Major Topic] NOT (Animals[Mesh] NOT (Animals[Mesh] AND Humans[Mesh])) AND (2000/1/1:3000/12/12[pdat]) AND (english[Filter]) | 29,677 |
| 3 | #1 OR #2 | 54,893 |
| 4 | (infant*[Title/Abstract] OR pedi*[Title/Abstract] OR paed*[Title/Abstract] OR child*[Title/Abstract] OR neonat*[Title/Abstract] OR newborn*[Title/Abstract] OR baby[Title/Abstract] OR babies[Title/Abstract] OR toddler*[Title/Abstract] OR preschool[Title/Abstract] OR pre-school[Title/Abstract]) NOT (Animals[Mesh] NOT (Animals[Mesh] AND Humans[Mesh])) AND (2000/1/1:3000/12/12[pdat]) AND (english[Filter]) | 1,108,557 |
| 5 | (infant[MeSH Major Topic] OR "child, preschool"[MeSH Major Topic]) NOT (Animals[Mesh] NOT (Animals[Mesh] AND Humans[Mesh])) AND (2000/1/1:3000/12/12[pdat]) AND (english[Filter]) | 28,029 |
| 6 | #4 OR #5 | 1,111,113 |
| 7 | (stool[Title/Abstract] OR fecal[Title/Abstract] OR faecal[Title/Abstract] OR gut[Title/Abstract] OR feces[Title/Abstract] OR faeces[Title/Abstract] OR  intestin*[Title/Abstract] OR gastro*[Title/Abstract]) NOT (Animals[Mesh] NOT (Animals[Mesh] AND Humans[Mesh])) AND (2000/1/1:3000/12/12[pdat]) AND (english[Filter]) | 322,049 |
| 8 | ("gastrointestinal microbiome"[MeSH Terms] OR "gastrointestinal tract"[MeSH Terms] OR feces[MeSH Terms]) NOT (Animals[Mesh] NOT (Animals[Mesh] AND Humans[Mesh])) AND (2000/1/1:3000/12/12[pdat]) AND (english[Filter]) | 223,933 |
| 9 | #7 OR #8 | 437,357 |
| 10 | #3 AND #6 AND #9 | 915 |

**Cochrane Central Register of Controlled Trials**

Date accessed: November 15, 2021

|  | **Search** | **Number Results Returned** |
| --- | --- | --- |
| 1 | ((antibiotic NEXT resistan*) OR (antimicrobial NEXT resistan*) OR (antibacterial NEXT resistan*) OR (anti-microbial NEXT resistan*) OR (anti-bacterial NEXT resistan*) OR resistome* OR (mobile NEXT genetic NEXT element*) OR (resistance NEXT gene*)):ti,ab,kw (Word variations have been searched) with Publication Year from 2000 to present, in Trials | 2795 |
| 2 | (infant* OR pedi* OR paed* OR child* OR neonat* OR newborn* OR baby OR babies OR toddler* OR preschool OR pre-school):ti,ab,kw (Word variations have been searched) with Publication Year from 2000 to present, in Trials | 177,760 |
| 3 | (stool OR fecal OR faecal OR gut OR feces OR faeces OR intestin* OR gastro*):ti,ab,kw (Word variations have been searched) with Publication Year from 2000 to present, in Trials | 74,585 |
| 4 | #1 AND #2 AND #3 | 128 |

**Search strategy peer review**

**Concept Map Overview:**

| **Concept** | **Keywords (tiab)** | **MesH (mesh)** |
| --- | --- | --- |
| Antibiotic | Antibiotic*, antibacterial*, anti-bacterial*, antimicrobial*, anti-microbial*, amoxicillin, penicillin*, ampicillin, ceph*, beta-lact*, azithromycin, macrolide*, tetracycline*, ciprofloxacin, augmentin, fluoroquinolone* | Anti bacterial Agents, Antibiotic Prophylaxis,  Macrolides, beta-Lactams, Tetracyclines, Fluoroquinolones |
| Antibiotic resistance genes | Antibiotic resistance, antimicrobial resistance, antibacterial resistance, anti-bacterial resistance, anti-microbial resistance, resistome*, mobile genetic element*, resistance gene* | Drug Resistance, Bacterial |
| Child | Infant*, pedi*, paed*, child*, neonat*, newborn*, baby, babies | Infant; Child, Preschool |
| Gut | Stool, fecal, faecal, gut, feces, intestin*, gastro* | Gastrointestinal Microbiome, Gastrointestinal Tract, Feces |

Note: One of the main interests in piloting this search strategy was to see if we needed to include “Antibiotic” terms or if they would all get captured in terms for “Antibiotic resistance genes”

**Web of Science**

Date Accessed October 29, 2021

Publication periods: 1/1/2000 – 1/1/3000

Refined by: Document Types: Articles or Proceedings Papers

Make sure you include the English limit

|  | **Topic Search (includes title, abstract, and keyword)** | **Results returned** | **Query Link** |
| --- | --- | --- | --- |
| 1 | TS=(“antibiotic*” OR “antibacterial*” OR “anti-bacterial*” OR “antimicrobial*” OR “anti-microbial*” OR “amoxicillin” OR “penicillin*” OR “ampicillin” OR “beta-lact*” OR “ceph*” OR “azithromycin” OR “macrolide*” OR “tetracycline*” OR “ciprofloxacin” OR “augmentin” OR “fluoroquinolone*”) | 585,850 | [Link](https://www.webofscience.com/wos/woscc/summary/0c3da42e-c881-4f2e-b60d-3465bff3627f-0fb7a0f4/relevance/1) |
| 2 | TS=(“antibiotic resistance” OR “antimicrobial resistance” OR “antibacterial resistance” OR “anti-bacterial resistance” OR “anti-microbial resistance” OR “resistome*” OR “mobile genetic element*” OR “resistance gene*”) | 91,809 | [Link](https://www.webofscience.com/wos/woscc/summary/4d1361dd-bfc7-4229-ad18-40195cd66cb3-0fb7a80f/relevance/1) |
| 3 | TS=(“infant*” OR “pedi*” OR “paed*” OR “child*” OR “neonat*” OR “newborn*” OR “baby” OR “babies”) | 1,642,624 | [Link](https://www.webofscience.com/wos/woscc/summary/4e072367-98c0-41a1-ad69-e018c4f3ed12-0fb7a9fb/relevance/1) |
| 4 | TS=(“stool” OR “fecal” OR “faecal” OR “gut” OR “feces” OR “ntestine*” OR “gastro*”) | 599,619 | [Link](https://www.webofscience.com/wos/woscc/summary/dcf36416-5685-4cfc-a84e-fe5b758fcdef-0fb7ac42/relevance/1) |
| 5 | 2 AND 3 AND 4 | 1,307 | [Link](https://www.webofscience.com/wos/woscc/summary/dafebe67-0f26-40cb-a127-9f94e2855c4c-0fb7b7bd/relevance/1) |
| 6 | 1 AND 5 | 1,283 | [Link](https://www.webofscience.com/wos/woscc/summary/64995de2-1dbf-40b1-a2b3-e590c33fdfb3-0fb7bb03/relevance/1) |

**Scopus**

Date Accessed October 29, 2021

Publication year > 1999

Limit to English

Limit to Article or Conference Paper

|  | **Search** | **Results returned** |
| --- | --- | --- |
| 1 | TITLE-ABS-KEY ( “antibiotic*”  OR  “antibacterial*”  OR  “anti-bacterial*”  OR  “antimicrobial*”  OR  “anti-microbial”  OR  “amoxicillin”  OR  “penicillin*”  OR  “ampicillin”  OR  “beta-lact*”  OR  “ceph*”  OR  “azithromycin”  OR  “macrolide*”  OR  “tetracycline*”  OR  “ciprofloxacin”  OR  “augmentin”  OR  “fluoroquinolone*” )  AND  PUBYEAR  >  1999  AND  ( LIMIT-TO ( DOCTYPE ,  “ar” )  OR  LIMIT-TO ( DOCTYPE ,  “cp” ) )  AND  ( LIMIT-TO ( LANGUAGE ,  “English” ) ) | 786,896 |
| 2 | TITLE-ABS-KEY ( “antibiotic resistance”  OR  “antimicrobial resistance”  OR  “antibacterial resistance”  OR  “anti-bacterial resistance”  OR  “anti-microbial resistance”  OR  “resistome*”  OR  “mobile genetic element*”  OR  “resistance gene*” )  AND  PUBYEAR  >  1999  AND  ( LIMIT-TO ( DOCTYPE ,  “ar” )  OR  LIMIT-TO ( DOCTYPE ,  “cp” ) )  AND  ( LIMIT-TO ( LANGUAGE ,  “English” ) ) | 132,143 |
| 3 | TITLE-ABS-KEY ( “infant*”  OR  “pedi*”  OR  “paed*”  OR  “child*”  OR  “neonat*”  OR  “newborn*”  OR  “baby”  OR  “babies” )  AND  PUBYEAR  >  1999  AND  ( LIMIT-TO ( DOCTYPE ,  “ar” )  OR  LIMIT-TO ( DOCTYPE ,  “cp” ) )  AND  ( LIMIT-TO ( LANGUAGE ,  “English” ) ) | 2,010,956 |
| 4 | TITLE-ABS-KEY ( “stool”  OR  “fecal”  OR  “faecal”  OR  “gut”  OR  “feces”  OR  “intestin*”  OR  “gastro*” )  AND  PUBYEAR  >  1999  AND  ( LIMIT-TO ( DOCTYPE ,  “ar” )  OR  LIMIT-TO ( DOCTYPE ,  “cp” ) )  AND  ( LIMIT-TO ( LANGUAGE ,  “English” ) ) | 768,519 |
| 5 | 2 AND 3 AND 4 | 2,380 |
| 6 | 1 AND 5 | 2,364 |

**PubMed**

Date Accessed: October 29, 2021

Dates searched: 2000 – 3000/12/12

Limit to English

Limit Human Only Studies

|  | **Search** | **Number Results Returned** |
| --- | --- | --- |
| 1 | (“antibiotic*”[Title/Abstract] OR “antibacterial*”[Title/Abstract] OR “anti-bacterial*”[Title/Abstract] OR “antimicrobial*”[Title/Abstract] OR “anti-microbial*” [Title/Abstract] OR “amoxicillin”[Title/Abstract] OR “penicillin*”[Title/Abstract] OR “ampicillin”[Title/Abstract] OR “ceph*”[Title/Abstract] OR “beta lact*”[Title/Abstract] OR “azithromycin”[Title/Abstract] OR “macrolide*”[Title/Abstract] OR “tetracycline*”[Title/Abstract] OR “ciprofloxacin”[Title/Abstract] OR “augmentin”[Title/Abstract] OR “fluoroquinolone*” [Title/Abstract]) | 236,671 |
| 2 | (“anti bacterial agents”[MeSH Terms] OR “antibiotic prophylaxis”[MeSH Terms] OR “macrolides”[MeSH Terms] OR “beta lactams”[MeSH Terms] OR “tetracycline”[MeSH Terms] OR “fluoroquinolones”[MeSH Terms]) | 227,549 |
| 3 | 1 OR 2 | 343,389 |
| 4 | (“antibiotic resistance”[Title/Abstract] OR “antimicrobial resistance”[Title/Abstract] OR “antibacterial resistance”[Title/Abstract] OR “anti-bacterial resistance”[Title/Abstract] OR “anti-microbial resistance” [Title/Abstract] OR “resistome*”[Title/Abstract] OR “mobile genetic element*”[Title/Abstract] OR “resistance gene*”[Title/Abstract]) | 32,661 |
| 5 | “drug resistance, bacterial”[MeSH Major Topic] | 29,582 |
| 6 | 4 OR 5 | 51,716 |
| 7 | (“infant*”[Title/Abstract] OR “pedi*”[Title/Abstract] OR “paed*”[Title/Abstract] OR “child*”[Title/Abstract] OR “neonat*”[Title/Abstract] OR “newborn*”[Title/Abstract] OR “baby”[Title/Abstract] OR “babies”[Title/Abstract]) | 1,099,753 |
| 8 | (“infant”[MeSH Major Topic] OR “child, preschool”[MeSH Major Topic]) | 27,954 |
| 9 | 7 OR 8 | 1,104,323 |
| 10 | (“stool”[Title/Abstract] OR “fecal”[Title/Abstract] OR “faecal”[Title/Abstract] OR “gut”[Title/Abstract] OR “feces”[Title/Abstract] OR “intestin*”[Title/Abstract] OR “gastro*”[Title/Abstract]) | 319,424 |
| 11 | (“gastrointestinal microbiome”[MeSH Major Topic] OR “gastrointestinal tract”[MeSH Major Topic] OR “feces”[MeSH Major Topic]) | 132,657 |
| 12 | 10 OR 11 | 383,788 |
| 13 | 6 AND 9 AND 12 | 747 |
| 14 | 3 AND 13 | 722 |

**Cochrane Central Register of Controlled Trials**

Date Accessed: October 29, 2021

Limit to English Only

CENTRAL Trials only Original publication year Between 2000 and present

Word variations have been searched

|  | **Search** | **Number Results Returned** |
| --- | --- | --- |
| 1 | (“antibiotic*” OR “antibacterial*” OR “anti-bacterial*” OR “antimicrobial*” OR “anti-microbial*” OR “amoxicillin*” OR “penicillin*” OR “ampicillin” OR “beta-lact*” OR “ceph*” OR “azithromycin” OR “macrolide*” OR “tetracycline*” OR “ciprofloxacin” OR “augmentin” OR “fluoroquinolone*”):ti,ab,kw | 37,044 |
| 2 | (“antibiotic resistance” OR “antimicrobial resistance” OR “antibacterial resistance” OR “anti-microbial resistance” OR “anti-bacterial resistance” OR “resistome*” OR “mobile genetic element*” OR “resistance gene*”):ti,ab,kw | 2,637 |
| 3 | (“infant*” OR “pedi*” OR “paed” OR “child*” OR “neonat*” OR “newborn*” OR “baby” OR “babies”):ti,ab,kw | 170,304 |
| 4 | (“stool” OR “fecal” OR “faecal” OR “gut” OR “feces” OR “intestin*” OR “gastro*”):ti,ab,kw | 38,017 |
| 5 | #2 AND #3 AND #4 | 96 |
| 6 | #1 AND #5 | 96 |

**PRESS peer review checklist**

| **Translation of the research question** | **Comments** |
| --- | --- |
| • Does the search strategy match the research question/PICO?  • Are the search concepts clear?  • Are there too many or too few PICO elements included?  • Are the search concepts too narrow or too broad?  • Does the search retrieve too many or too few records?  • Are unconventional or complex strategies explained? | Yes  Yes  No  No  No  n/a |
| **Boolean operators** | **Comments** |
| • Are AND/OR used correctly?  • Is the use of nesting with brackets appropriate and effective for the search?  • If NOT is used, is this likely to result in any unintended exclusions? | Yes  n/a  n/a |
| **Proximity operators** | **Comments** |
| • Are proximity operators used correctly?  • Could precision be improved by using proximity operators (e.g., adjacent, near) or phrase-searching instead of AND?  • Is the width of proximity operators suitable (e.g., adj2/adj5)? | Need to add them for Cochrane truncated phrases  Doesn’t see necessary  n/a |
| **Subject headings** | **Comments** |
| - Are the subject headings relevant? - Are any relevant subject headings missing (e.g., previous index terms)? - Are any subject headings too broad or too narrow? - Are subject headings exploded where necessary? - Are all exploded headings relevant? - Are major headings used? If so, is there adequate justification? - Are subheadings used appropriately? - Are subheadings attached to subject headings? (Floating subheadings may be preferred.) - Are both subject headings and terms in free text (see below) used for each concept? | Yes  No  Is there any reason to go with the broader “tetracyclines”[MeSH] rather than "tetracycline"[MeSH  Yes  Yes  Yes—the subject heading for the Gut concept  n/a  n/a  yes |
| **Text word searching** | **Comments** |
| - Can you think of any other relevant synonyms, related terms and antonyms been searched? - Are acronyms and abbreviations used appropriately? Do they capture irrelevant material? - Is truncation used appropriately (i.e. truncated at the correct place)? - Are the keywords specific enough or too broad? - Have the appropriate fields been searched? - Should any long phrases be broken into several shorter search statements? | See suggestions in comments for WoS search  None used  Yes  Appropriate  Yes  Appropriate |
| **Spelling** | **Comments** |
| - Are there any spelling errors? - Are alternate spellings included (e.g., UK versus US)? | No  See suggestion for faeces |
| **Search Limits and Filters** | **Comments** |
| - Are limits and filters used appropriately and are they relevant given the research question? - Are any potentially helpful limits or filters missing? - Can any limits or filters be added or taken away? - Are sources cited for the filters used? | - I didn’t see a justification for the 2000 cutoff - for PubMed I recommend using the Humans limit hedge: FinalResult NOT ("Animals"[Mesh] NOT ("Animals"[Mesh] AND "Humans"[Mesh])) - Limiting to articles or proceeding in WoS and Scopus will mean you don’t find systematic reviews. Is that ok?   No  No  n/a |
| **Search line numbers** | **Comments** |
| - Are there incorrect line combinations (e.g. errors in an AND or OR statements)? - Are there any orphan lines? | Make sure to use #symbol in front of set numbers for WoS and Scopus  No |
| **Strategy adapted to each database** | **Comments** |
| - Have all relevant databases been identified? - Has the search strategy been adapted for each database? | Yes  Yes |
| **Additional Comments** | |
| When recording the searches, make sure to record the searches as run. I was unable to evaluate the use of limits in several of the searches because they were not included in the captured search strategies.  PRESS was completed by Pamela Bagley on November 5, 2021. | |

Adapted from TABLE 9: PRESS 2015 EVIDENCE-BASED CHECKLIST at <https://www.cadth.ca/sites/default/files/pdf/CP0015_PRESS_Update_Report_2016.pdf>

**Example data extraction form**

## General Information

| **Question** | **Answer** |
| --- | --- |
| 1. **Study ID** *(surname of first author and year first full report of study was published e.g. Smith 2001)* |  |
| 1. **Initials of person extracting data** |  |
| 1. **Title** |  |
| 1. **Journal** |  |
| 1. **Notes:** |  |

## Study overview

|  | **Answer (descriptions should be as stated in report/paper)** | **Location in text**  *(pg & ¶/fig/table)* |
| --- | --- | --- |
| 1. **Aim of study** |  |  |
| 1. **Key conclusions of study authors** |  |  |
| 1. **Randomized study**   *(Yes/no/unclear)* |  |  |
| 1. **Study design** *(Randomized controlled trial, randomized cluster trial, prospective cohort study, retrospective cohort study, ambidirectional cohort study, case-control study, ecologic, quasi-experimental, unclear)* |  |  |
| 1. **Start and end date(s)** |  |  |
| 1. **Notes:** |  |  |

## Population and setting

|  | | **Description**  *Include comparative information for each group (i.e. intervention and controls) if available* | **Location in text**  *(pg & ¶/fig/table)* |
| --- | --- | --- | --- |
| 1. **Population description**   *(from which study participants are drawn)* | |  |  |
| 1. **Total no. participants** *(for randomized studies, total undergoing randomization)* | |  |  |
| 1. **Setting**   *(including location/country and social context)* | |  |  |
| 1. **Inclusion criteria** | |  |  |
| 1. **Exclusion criteria** | |  |  |
| 1. **Method/s of recruitment of participants** | |  |  |
| 1. **Notes:** |  | | |

## Intervention groups

**Note**: We are primarily interested in exposure-outcome associations related to antibiotics given directly to infants and their antibiotic resistance genes. Thus, there is no need to document additional exposure or outcome groups. We are not primarily interested in antibiotics given to mothers before birth or antibiotics given to mothers during delivery and labor (sometimes called intrapartum antibiotic prophylaxis or perinatal antibiotic exposure).

*Copy and paste table for each intervention/comparison group*

### Intervention Group 1

|  | | **Description as stated in report/paper** | **Location in text**  *(pg & ¶/fig/table)* |
| --- | --- | --- | --- |
| 1. **Group name** *(e.g., any antibiotic exposure, number/frequency exposures, any antibiotic exposure for a particular antibiotic, number/frequency of antibiotic exposure for a particular type of antibiotic)* | |  |  |
| 1. **No. randomised or in group**   *(specify whether no. people or clusters)* | |  |  |
| 1. **Antibiotic(s) considered** (any; unless they were only interested in specific exposures) | |  |  |
| 1. **Antibiotic exposure(s) as stated** | |  |  |
| 1. ***Class of antibiotic exposure(s)** | |  |  |
| 1. ****Indicated reason(s) for antibiotic exposure** | |  |  |
| 1. **Duration of treatment period** *(time frame in which intervention was considered in the study)* | |  |  |
| 1. **Timing**   *(e.g. frequency, duration of each antibiotic episode episode)* | |  |  |
| 1. **Delivery**   *(oral, intravenous, etc.)* | |  |  |
| 1. **Antibiotic documentation** (medical record review, parental recall, interview, etc.) | |  |  |
| 1. **Notes:** |  | | |

## *Class of antibiotic(s) assessed:

-Macrolides (includes azithromycin)

-Beta-lactams (includes cephalosporins, cefazolin, cefdinir, cephalexin, carbapenems, penicillins, amoxicillin, augmentin, ampicillin, meropenem)

-aminoglycosides (gentamicin, streptomycin)

-tetracyclines

-fluoroquinolones (includes ciprofloxacin)

-vancomycin

-clindamycin

-sulfanilamides (almost any drug with sulfa including sulfamethoxazole/trimethoprim)

-nitroimidazoles (includes metronidazole)

****Indicated reason(s) for antibiotic exposure:**

-otitis media (ear infection)

-lower respiratory tract infections (pneumonia, bronchitis, bronchiolitis, tonsillitis, laryngitis)

-upper respiratory tract infections (common cold, sinus infections, tonsillitis, laryngitis)

-prophylactic exposure (including part of mass administration)

-complications at birth (includes sepsis or pre-term birth)

## Outcomes

|  | |  |  |
| --- | --- | --- | --- |
|  | | **Description as stated in report/paper** | **Location in text**  *(pg & ¶/fig/table)* |
| 1. **Database (version) of ARGs used in metagenomes** *(e.g., CARD 3.1.4)* | |  |  |
| 1. **Sequencing tool used to measure antibiotic resistance genes** *(e.g., whole-genome sequencing, shotgun sequencing, quantitative PCR)* | |  |  |
| 1. **Sequencing platform used** *(e.g., Illumina NovaSeq, Illumina MiSeq, etc.)* | |  |  |
| 1. **Single or paired-end sequencing?** | |  |  |
| 1. **Target sequencing depth** | |  |  |
| 1. **Reported sequencing depth** *(mean or median)* | |  |  |
| 1. **Outcome name** | | **RICHNESS (Number of unique types of resistance genes)** |  |
| 1. **Time point(s) reported** | |  |  |
| 1. **Unit of measurement** | |  |  |
| 1. **Outcome name** | | **ALPHA DIVERSITY (combo of richness & evenness)** |  |
| 1. **Time points reported** | |  |  |
| 1. **Unit of measurement** (e.g., Shannon, Simpson, Chao) | |  |  |
| 1. **Outcome name** | | **RESISTANCE LOAD (overall resistance gene load)** |  |
| 1. **Time points reported** | |  |  |
| 1. **Unit of measurement** | |  |  |
| 1. **Outcome name** | | **PRESENCE/ABSENCE INDIVIDUAL RESISTANCE GENES** |  |
| 1. **Time points reported** | |  |  |
| 1. **Unit of measurement** | |  |  |
| 1. **Notes:** |  | | |

## Results

**Note**: We are primarily interested in exposure-outcome associations related to antibiotics and antibiotic resistance genes. Thus, there is no need to document additional exposure or outcome groups. Papers frequently will describe richness and alpha diversity as related to microbial diversity (e.g. the composition of bacterial taxa present) – no need to capture that information.

### **Presence/absence of specific antibiotic resistance genes:**

Please download [THIS FILE](https://docs.google.com/spreadsheets/d/1mvdfNX5imZRp1ARV2mCDmRgRGSEe8oYBuxnzllNXbmI/edit?usp=sharing) that lists the names and, if possible, classes of antibiotic resistance genes that with gene presence/absence by intervention group(s). This may include searching supplemental files. If no information about antibiotic resistance gene data by intervention group(s) is available, please write that you checked this below and could not identify that they directly assessed gene presence/absence by antibiotic exposure group.

|  |  | **Description as stated in report/paper** | **Location in text**  *(pg & ¶/fig/table)* |
| --- | --- | --- | --- |
| 1. **Number of antibiotic resistance genes screened** | |  |  |
| 1. **Number of antibiotic resistance genes identified in at least one sample** | |  |  |
| 1. **Notes** | |  | |

### For randomised or non-randomised trial - RICHNESS

|  | | | **Description as stated in report/paper** | | | | | | | | **Location in text**  *(pg & ¶/fig/table)* | |
| --- | --- | --- | --- | --- | --- | --- | --- | --- | --- | --- | --- | --- |
| 1. **Subgroup** | | |  | | | | | | | |  | |
| 1. **Time point**   *(specify whether from start or end of intervention)* | | |  | | | | | | | |  | |
| 1. **Post-intervention or change from baseline?** | | |  | | | | | | | |  | |
| 1. **Results**   *Note whether:*  *post-intervention OR*  *change from baseline*  *And whether*  *Adjusted OR*  *Unadjusted* | | **Intervention** | | | | | **Comparison** | | | |  |  |
|  |  | Mean or median | | SD, Pval, CI, or other variance | No. participants | | Mean or median | | SD, Pval, CI, or other variance | No. participants |  |  |
|  |  |  | |  |  | |  | |  |  |  |  |
| 1. **Baseline data** | | **Intervention** | | | | | **Comparison** | | | |  |  |
|  |  | Mean or median | | SD, Pval, CI, or other variance | No. participants | | Mean or median | | SD, Pval, CI, or other variance | No. participants |  |  |
|  |  |  | |  |  | |  | |  |  |  |  |
| 1. **If adjusted, covariates adjusted for** *(e.g., delivery method, breast vs. formula feeding, siblings, etc)* | | |  | | | | |  | | |  | |
| 1. **No. missing participants and reasons** | | |  | | | | |  | | |  | |
| 1. **No. participants moved from other group and reasons** | | |  | | | | |  | | |  | |
| 1. **Any other results reported** | | |  | | | | | | | |  | |
| 1. **Statistical methods used and appropriateness of these methods**   *(e.g. adjustment for correlation)* | | |  | | | | | | | |  | |
| 1. **Reanalysis required?**   *(if yes, specify why)* | | | *Yes/No/Unclear* | | |  | | | | |  | |
| 1. **Reanalysis possible?** | | | *Yes/No/Unclear* | | |  | | | | |  | |
| 1. **Reanalysed results** | | |  | | | | | | | |  | |
| 1. **Notes:** |  | | | | | | | | | | | |

### For randomised or non-randomised trial – ALPHA DIVERSITY (Richness & evenness)

|  | | | **Description as stated in report/paper** | | | | | | | | **Location in text**  *(pg & ¶/fig/table)* | |
| --- | --- | --- | --- | --- | --- | --- | --- | --- | --- | --- | --- | --- |
| 1. **Subgroup** | | |  | | | | | | | |  | |
| 1. **Time point**   *(specify whether from start or end of intervention)* | | |  | | | | | | | |  | |
| 1. **Post-intervention or change from baseline?** | | |  | | | | | | | |  | |
| 1. **Results**   *Note whether:*  *post-intervention OR*  *change from baseline*  *And whether*  *Adjusted OR*  *Unadjusted* | | **Intervention** | | | | | | **Comparison** | | |  |  |
|  |  | Mean or median | | SD, Pval, CI, or other variance | No. participants | | | Mean or median | SD, Pval, CI, or other variance | No. participants |  |  |
|  |  |  | |  |  | | |  |  |  |  |  |
| 1. **Baseline data** | | **Intervention** | | | | | | **Comparison** | | |  |  |
|  |  | Mean or median | | SD, Pval, CI, or other variance | No. participants | | | Mean or median | SD, Pval, CI, or other variance | No. participants |  |  |
|  |  |  | |  |  | | |  |  |  |  |  |
| 1. **No. missing participants and reasons** | | |  | | | |  | | | |  | |
| 1. **No. participants moved from other group and reasons** | | |  | | | |  | | | |  | |
| 1. **Any other results reported** | | |  | | | | | | | |  | |
| 1. **Statistical methods used and appropriateness of these methods**   *(e.g. adjustment for correlation)* | | |  | | | | | | | |  | |
| 1. **Reanalysis required?**   *(if yes, specify why)* | | | *Yes/No/Unclear* | | |  | | | | |  | |
| 1. **Reanalysis possible?** | | | *Yes/No/Unclear* | | |  | | | | |  | |
| 1. **Reanalysed results** | | |  | | | | | | | |  | |
| 1. **Notes:** |  | | | | | | | | | | | |

### For randomised or non-randomised trial – RESISTANCE LOAD

|  | | | **Description as stated in report/paper** | | | | | | | | **Location in text**  *(pg & ¶/fig/table)* | |
| --- | --- | --- | --- | --- | --- | --- | --- | --- | --- | --- | --- | --- |
| 1. **Subgroup** | | |  | | | | | | | |  | |
| 1. **Time point**   *(specify whether from start or end of intervention)* | | |  | | | | | | | |  | |
| 1. **Post-intervention or change from baseline?** | | |  | | | | | | | |  | |
| 1. **Results**   *Note whether:*  *post-intervention OR*  *change from baseline*  *And whether*  *Adjusted OR*  *Unadjusted* | | **Intervention** | | | | | | **Comparison** | | |  |  |
|  |  | Mean or median | | SD, Pval, CI, or other variance | No. participants | | | Mean or median | SD, Pval, CI, or other variance | No. participants |  |  |
|  |  |  | |  |  | | |  |  |  |  |  |
| 1. **Baseline data** | | **Intervention** | | | | | | **Comparison** | | |  |  |
|  |  | Mean or median | | SD, Pval, CI, or other variance | No. participants | | | Mean or median | SD, Pval, CI, or other variance | No. participants |  |  |
|  |  |  | |  |  | | |  |  |  |  |  |
| 1. **No. missing participants and reasons** | | |  | | | |  | | | |  | |
| 1. **No. participants moved from other group and reasons** | | |  | | | |  | | | |  | |
| 1. **Any other results reported** | | |  | | | | | | | |  | |
| 1. **Statistical methods used and appropriateness of these methods**   *(e.g. adjustment for correlation)* | | |  | | | | | | | |  | |
| 1. **Reanalysis required?**   *(if yes, specify why)* | | | *Yes/No/Unclear* | | |  | | | | |  | |
| 1. **Reanalysis possible?** | | | *Yes/No/Unclear* | | |  | | | | |  | |
| 1. **Reanalysed results** | | |  | | | | | | | |  | |
| 1. **Notes:** |  | | | | | | | | | | | |

## Other information

| 1. **Additional reports assessed with current study** |  |
| --- | --- |
| 1. **Additional references to check from study** | **(For Becky)** |
| 1. **Notes:** |  |

**Amendments to ROBINS-I criteria**

Our potential bias assessment focused on the association between antibiotics and resistome outcomes. We jointly considered all resistome outcomes due to the lack of variance in collection and measurement of stool samples. There is currently no gold standard for measuring the resistome which would make it challenging to assess the validity of metrics used (e.g., alpha diversity, abundance, etc.), so all resistome outcomes were considered jointly for the bias assessment.

The comments below were considered as an amendment to the rubric provided with the ROBINS-I tool. Some studies were not primarily designed to assess this particular association so some amended guidelines were created.

1. **Bias due to confounding**

- Due to the inherent nature of unmeasured confounding in almost all microbiome and resistome studies, we accounted for this while assessing risk of bias due to confounding.
- Low:
  - The study controlled for multiple confounders of interest.
  - The study had a decision system or strong rationale for determining confounders.
  - The study is unlikely to have major amounts of residual confounding beyond what is expected due to expected variation in microbiome/resistome studies.
- Moderate:
  - The study controlled or considered at least one major confounder. This could be through stratification or restriction in addition to adjustment.
  - There is a low amount of expected residual confounding.
  - The study has some rationale for confounders included or not included in the analysis.
- Serious:
  - There have been no attempts to control for confounding in the analysis of the exposure-outcome of interest and no information related to why they did not control for confounding.
  - There is potential for residual confounding.
  - Insufficient metadata is provided to evaluate possible confounders of the exposure-outcome association.
- Critical:
  - There is no mention of any confounding factors or ways to control them.
  - The study almost certainly has unmeasured residual confounding.
- No information:
  - The study did not directly assess the exposure-outcome association so bias due to confounding could not be evaluated.

1. **Bias in selection of participants into the study**

- Low:
  - No noted or possible selection bias or loss to follow-up bias.
- Moderate:
  - There is some possibility for selection bias or loss to follow-up bias but it’s not expected to lead to major differences in the association.
- Serious:
  - There is a strong possibility of selection bias or loss to follow-up bias in the study that would lead to major differences in the exposure-outcome association. This was not considered or adjusted for in the analyses.
- Critical:
  - Selection bias or loss to follow-up bias is critical and could not be adjusted for.

1. **Bias in the classification of interventions**

- Low:
  - The intervention status was clearly defined and based on information collected primarily at the time of collection.
  - If the study provides clear information on the number of days an antibiotic is given per child (e.g., a longitudinal plot) and other detailed information about antibiotic exposure is provided, a “Low” rating is reasonable even if there is some element of retrospective review.
- Moderate:
  - The intervention status is clearly defined but antibiotic information was primarily determined retrospectively (e.g., only via parental recall or interview).
- Serious:
  - The intervention status is not well defined.
- Critical:
  - An extremely high amount of misclassification is likely (recall bias is severe or there were inconsistencies in how antibiotic exposures were measured).

1. **Bias due to deviations from intended interventions**

The ROBINS-I guide was followed in entirety here.

1. **Bias due to missing data**

The ROBINS-I guide was followed in entirety here.

1. **Bias in measurement of outcomes**

- Low:
  - Timepoints and measurement of stool samples were the same across antibiotic exposure groups.
  - No clear differences in measurement of ARGs by group.
- Moderate:
  - The methods of outcome measurement were slightly different across groups (e.g., more samples have been collected in the antibiotic exposed group) but this is unlikely to have a significant effect on the exposure-outcome association.
- Serious:
  - The methods of outcome measurement were significantly different across groups (e.g., the timepoints for measurement were significantly different across groups) which may have an impact on the exposure-outcome association.
- Critical:
  - The methods of outcome measurement were so significantly different across groups that it definitely had an impact on the exposure-outcome association.

1. **Bias in selection of the reported result**

- Low:
  - There is clear evidence that all planned analyses correspond to all published analyses.
  - This typically would necessitate that the study had an established protocol or statistical analysis plan available.
- Moderate:
  - There seems to be internal and external consistency of the planned and reported analyses. This includes commentary on why specific analyses were selected.
  - There is no indication suggesting selection of the reported results from among multiple analyses based on what would be statistical significance/greatest effect.
  - There is no indication of selection of a specific cohort to present better looking results.
- Serious:
  - Outcomes are defined in different ways internally or across different publications from the study
  - OR there is a high risk of selective reporting from analyses
  - OR this cohort is a subgroup selected from a larger study for analysis and appears to be reported on the basis of the results.
- Critical:
  - The results are underreported and there is strong suspicion of selective reporting of results.

**Overall bias**

The ROBINS-I guide was followed in entirety here.

|  | **No. of studies** | **Study designs** | **Risk of bias** | **Inconsistency** | **Indirectness** | **Imprecision** | **Other** | **Quality** | **Importance** |
| --- | --- | --- | --- | --- | --- | --- | --- | --- | --- |
| Overall gene resistance load | 8 | RCTs and observational studies | Serious risk of bias | No serious inconsistency | No serious indirectness | Serious imprecision, lack of confidence intervals | Reporting bias, publication bias suspected, evidence of dose dependent effect | Low quality | Critical |
| Resistome alpha diversity | 9 | RCTs and observational studies | Serious risk of bias | Serious inconsistency, driven most likely by population age, antibiotic exposure frequency, and time since antibiotic exposure | No serious indirectness | Serious imprecision, lack of confidence intervals | Reporting bias, publication bias suspected | Very low quality | Important |

**GRADE: Quality of evidence for resistome outcomes**
